# Comparison of L-Carnitine and L-Carnitine HCL salt for targeted lung treatment of pulmonary hypertension (PH) as inhalation aerosols: Design, comprehensive characterization, *in vitro* 2D/3D cell cultures, and *in vivo* MCT-Rat model of PH

**DOI:** 10.1016/j.pupt.2021.101998

**Published:** 2021-02-05

**Authors:** Maria F. Acosta, Priya Muralidhran, Michael D. Abrahamson, Carissa L. Grijalva, Megan Carver, Haiyang Tang, Christina Klinger, Jeffrey R. Fineman, Stephen M. Black, Heidi M. Mansour

**Affiliations:** a The University of Arizona College of Pharmacy, Skaggs Pharmaceutical Sciences Center, 1703 E. Mabel St, 85721, Tucson, AZ, USA; b The University of Arizona College of Engineering, Department of Biomedical Engineering, 1209 East 2nd Street, Room 100, Tucson, AZ, 85719, USA; c The University of Arizona College of Medicine, Department of Medicine, Division of Translational and Regenerative Medicine, 501 N Campbell Ave, 85724, Tucson, AZ, USA; d The University of Arizona College of Medicine, Department of Medicine, Center for Lung Vascular Pathobiology, 501 N Campbell Ave, 85724, Tucson, AZ, USA; e University of California San Francisco School of Medicine, Department of Pediatrics, 1975 Fourth St, Fourth Floor, 94158, San Francisco, CA, USA; f University of California San Francisco Benioff Children’s Hospital, San Francisco, CA, USA; g University of California San Francisco Cardiovascular Research Institute, San Francisco, CA, USA; h The University of Arizona College of Medicine, Department of Physiology, 501 N Campbell Ave, 85724, Tucson, AZ, USA; i The University of Arizona, BIO5 Institute, 1657 E Helen St, 85719, Tucson, AZ, USA

**Keywords:** Crystallinity, Respiratory drug delivery, Targeted drug delivery, Monocrotaline, Drug development, Inhalation aerosol medicine, Pulmonary hypertension

## Abstract

Disrupted L-Carnitine (L-Car) homeostasis has been implicated in the development of pulmonary hypertension (PH). L-Car has been administered orally and intravenously causing systemic side effects. To the authors’ knowledge, there are no reports using L-Car or L-Car HCl as an inhaled aerosol through the respiratory route in a targeted manner either from dry powder inhaler (DPI) or liquid delivery system. The purpose of the comprehensive and systematic comparative study between L-Car and L-Car HCl salt was to design and develop dry powder inhalers (DPIs) of each. This was followed by comprehensive physicochemical characterization, *in vitro* cell viability as a function of dose on 2D human pulmonary cell lines from different lung regions and *in vitro* cell viability on 3D small airway epithelia human primary cells at the air-liquid interface (ALI). In addition *in vitro* transepithelial electrical resistance (TEER) in air-interface culture (AIC) conditions on 2D human pulmonary cell line and 3D small airway epithelia human primary cells was carried out. *In vitro* aerosol dispersion performance using three FDA-approved human DPI devices with different device properties was also examined. Following advanced spray drying under various conditions, two spray drying pump rates (low and medium) were found to successfully produce spray-dried L-Car powders while four spray drying pump rates (low, medium, medium-high, and high) all resulted in the production of spray-dried L-Car HCl powders. Raw L-Car and L-Car HCl were found to be crystalline. All SD powders retained crystallinity following spray drying and polymorphic interconversion in the solid-state was identified as the mechanism for retaining crystallinity after the advanced spray drying process. All SD powders aerosolized readily with all three human DPI devices. However, the *in vitro* dispersion parameters for the SD powders was not conducive for *in vivo* administration to rats in DPIs due to hygroscopicity and nanoaggreation. *In vivo* rat studies were successfully accomplished using inhaled liquid aerosols. Safety was successfully demonstrated *in vivo* in healthy Sprague Dawley rats. Furthermore, therapeutic efficacy was successfully demonstrated *in vivo* in the monocrotaline (MCT)-rat model of PH after two weeks of daily L-Car inhalation aerosol treatment.

## 1. Introduction

Pulmonary hypertension (PH) is diagnosed when the mean pulmonary artery pressure (PAP) is more than 25 mmHg at rest [1]. This disease principally targets the endothelium of pulmonary arteries resulting in vasoconstriction and an intense vascular remodeling [2,3]. This vasoconstriction, occurs, at least in part, due to an imbalance in the production of vasodilators and vasoconstrictors [2]. Oxidative stress is also involved in the development of PH. Oxidative stress arises when there is an excessive production or reduced clearance of reactive oxygen species (ROS) or a reduction in the ability of the cell to correct the redox state [2]. The mitochondria are also involved in the production of ROS (e.g. superoxide and hydrogen peroxide). Mitochondrial dysfunction is directly linked to PH [4]. Therapeutic strategies that reduce ROS levels are potential treatments for PH and other diseases where ROS levels are increased [2].

L-Carnitine (β-hydroxy-γ−4-n-trimethylaminobutyric acid) (L-Car) is a cofactor required for transport of long-chain fatty acids into the mitochondrial matrix, where they undergo β-oxidation for cellular energy production. L-carnitine (L-Car) is indispensable for energy metabolism and mitochondrial function in the myocardium. Although L-Car deficiency has been implicated in development of left ventricular failure, little is known about the role of L-carnitine in right ventricular failure in PH [5,6]. In addition, studies suggest that L-Car has an *anti*--peroxidative effect on several tissues, which may account for its beneficial effect in oxidant-induced injury, making L-Car an excellent candidate for PH therapy [5,6]. It has been demonstrated that supplementation with oral L-Car reduces superoxide production and averts endothelial dysfunction in lambs with early forms of PH [7]. Unfortunately, oral supplementation of L-Car has been correlated with the development of atherosclerosis via its metabolism by the gut microbiome [7]. Pulmonary drug delivery [8,9] would bypass this potential pathologic issue. Nebulizer liquid aerosol delivery [10,11], soft-mist inhalers [12–14], pressurized metered dose inhalers (pMDIs) [15–17], and dry powder inhalers (DPIs) [18–21] are among the different types of inhaler devices that can be used. Currently, inhaled liquid aerosols by nebulizer devices are used in the currently FDA-approved marketed pharmaceutical inhalation products indicated in the treatment of PH [10]. L-Car has been administered orally and intravenously but not delivered in respirable form [7]. Thus, the purpose of the comprehensive and systematic comparative study between L-Car and L-Car HCl salt was to design and develop dry powder inhalers (DPIs) using advanced organic solution spray drying in closed-mode using four spray drying pump rates (i.e. low, medium, medium-high, and high) of each. Three FDA-approved human DPI devices with different device properties were used with the Next-Generation Impactor™ (NGI) to examine the aerosol dispersion properties as DPIs. Comprehensive physicochemical characterization was carried out along with *in vitro* cell viability as a function of dose on various 2D human pulmonary cell lines from different lung regions, *in vitro* cell viability on 3D small airway epithelia human primary cells at the air-liquid interface (ALI), *in vitro* transepithelial electrical resistance (TEER) in air-interface culture (AIC) conditions, as well as *in vitro* aerosol dispersion performance. *In vivo* rat studies were also successfully carried using inhaled liquid aerosols. Safety of inhaled liquid L-Car aerosols was successfully demonstrated *in vivo* in healthy Sprague Dawley rats. Furthermore, therapeutic efficacy and PH reversal was demonstrated in the MCT-rat model of PH using daily two weeks of daily L-Car inhalation aerosol treatment 14-days after MCT exposure. To the authors’ knowledge, this is the first to report of aerosolized L-Car being an effect therapy for PH.

## 2. Experimental: materials & methods

### 2.1. Materials

**Fig. 1.**
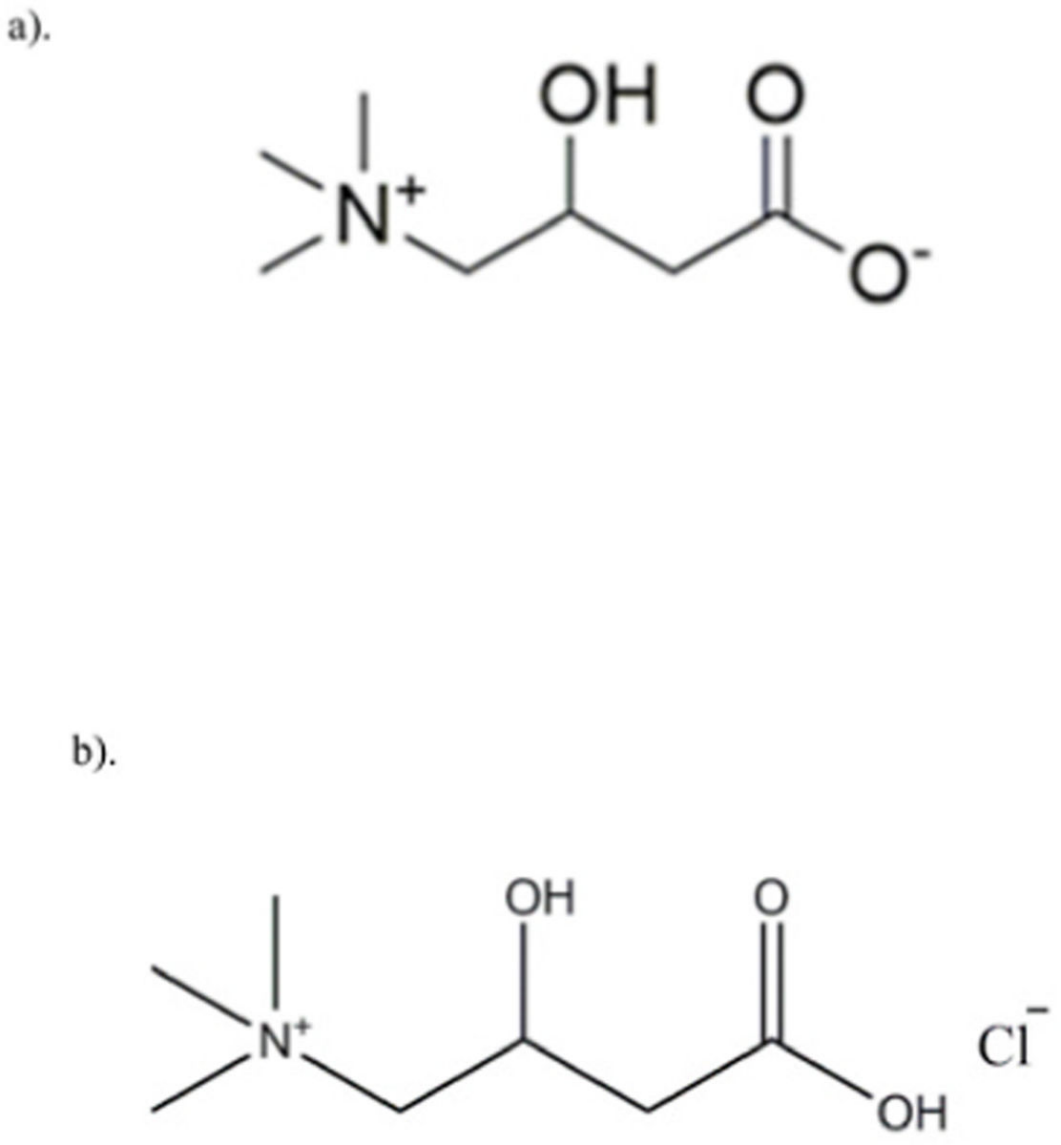
Chemical structures of: a). L-Carnitine (L-Car) and b). L-Carnitine HCl (L-Car HCl) (ChemDraw™ Ultra Ver. 15.0.; CambridgeSoft, Cambridge, MA).

Raw L-Carnitine (L-Car), 99+% purity (C_7_H_15_NO_3_; MW: 161.199 g/mol) powder was obtained from ACROS Organics™ (New Jersey, New Jersey). Raw L-Carnitine.HCl (L-Car.HCl) powder with < 98% purity (C_7_H_16_ClNO_3_: MW: 197.66) was obtained from Sigma-Aldrich®, Inc (St. Louis, MO). Both chemical structures are shown Fig. 1 (ChemDraw Ultra™ Ver. 15.0.; CambridgeSoft®, Cambridge, MA). Methanol (HPLC grade, ACS –certified grade, purity 99.9%) was obtained from Fisher Scientific™ (Fair Lawn, NJ). HYDRANAL®-Coulomat AD was from Sigma-Aldrich®, Inc (St. Louis, MO). Monocrotaline (MCT) powder was obtained from Sigma-Aldrich® (St. Louis, MO). Raw L-Car and L-Car.HCl were stored in sealed glass desiccators over indicating Drierite/Drierite™ desiccant at −20 °C under ambient pressure. Other chemicals were stored under room conditions. Ultra-high purity (UHP) nitrogen gas from (Cryogenics and gas facility, The University of Arizona, Tucson, AZ) was used.

Human pulmonary cell lines for different lung regions, A549 (ATCC® CCL-185™), NCI-H358 (ATCC® CRL-5807™), Calu-3 (ATCC® HTB-55™), and NCI-H441 (ATCC® HTB-174™) were purchased from the American Type Culture Collection ATCC® (Manassas, VA). Cell culture medium, Dulbecco’s modified Eagle’s medium (DMEM) Advanced 1X and RPMI 1640 were purchased from Gibco® by Life Technologies (Thermo Fisher Scientific Inc, Waltham, MA); Eagle’s Minimum Essential Medium (EMEM) (ATCC® 30 2003™) was purchased from the ATCC®. Supplements, Fetal Bovine Serum (FBS), Pen-Strep, Fungizone®, L-Glutamine, and Insulin-transferrin-selenium were obtained from Gibco® by Life Technologies (Thermo Fisher Scientific Inc, Waltham, MA); Dexamethasone was obtained from Sigma-Aldrich®, Inc (St. Louis, MO).

SmallAir™ (Epithelix, Geneva, Switzerland) is a unique 3D human small airway epithelia comprised of human pulmonary primary cells reconstituted *in vitro* with corresponding SmallAir™ special growth media (Epithelix, Geneva, Switzerland). The special growth media is serum-free and contains growth factors and phenol red.

For the *in vivo* animal studies, 3-week old Sprague Dawley male rats with a body weight in the range of 260g–315g per rat upon receipt were purchased from Charles River Laboratories International Inc. (Wilmington, MA). Rats were housed in the University of Arizona Animal Care (UAC) Facility for at least 1 week for acclimination prior to the initiation of experiments. Animals were kept in a 12-h light/dark cycle at an ambient temperature of 22 °C and received standard rodent food and water ad libitum. All experimental procedures were approved by the Institutional Animal Care and Use Committee (IACUC) at The University of Arizona.

### 2.2 Methods

#### 2.2.1. Preparation of SD particles by organic solution advanced spray drying in closed-mode

A Büchi B-290 Mini Spray Dryer with a high performance cyclone in close mode using UHP dry nitrogen as the atomizing gas and connected to the B-295 Inert Loop (Büchi Labortechnik AG, Flawil, Switzerland), as previously reported [22,23], was used to synthesize the dry particles. The feed solutions were prepared by dissolving either L-Car or L-Carnitine HCl in methanol solvent. All spray drying was conducted in closed-mode using nitrogen gas as the atomizing gas and also the drying gas to prevent the ingress of any oxygen nor moisture. The feed solution concentration was 0.5% w/v in methanol. Four spray drying pump rates (PRs) were tested which were 25% PR (low), 50% PR (medium), 75% PR (medium-high) and the maximum PR which is 100% PR (high). The other spray drying conditions such as the drying gas atomization rate (670 L/h at 35 mmHg), the aspiration rate (35 m3/h at 100% rate) and the inlet temperature (150 °C) were maintained constant during all the experiments. Table 1 lists the spray drying conditions for both L-Car and L-Car HCl. The corresponding outlet temperatures are summarized in Table 2. The stainless-steel nozzle diameter was 0.7 mm. The SD particles were separated from the nitrogen drying gas in the high-performance cyclone and collected in the small sample collector. All SD powders were carefully stored in sealed glass vials placed in sealed glass desiccators over indicating Drierite/Drierite™ desiccant at −20 °C.

#### 2.2.2. Scanning electron microscopy

Scanning electron microscopy (SEM) was conducted using a FEI Inspect S microscope (FEI, Brno, Czech Republic), using conditions similar to those previously reported [22,23]. Several magnification levels were used.

#### 2.2.3. Particle sizing and size distribution using SEM micrographs

The mean size, standard deviation, and size range were determined using SigmaScan™ Pro 5.0.0 (Systat, Inc., San Jose, CA) based on their scanning electron micrographs using a similar procedure previously reported [23,24].

#### 2.2.4. X-ray powder diffraction (XRPD)

A PANalytical X’pert diffractometer (PANalytical Inc., Westborough, Massachusetts) equipped with a programmable incident beam slit and an X’Celerator Detector, using conditions similar to those previously reported [22,23], was utilized to measure the degree of long-range molecular order (crystallinity) of all powders. The x-ray radiation used was Ni-filtered Cu Kα (45 kV, 40 Ma, and λ = 1.5418 Å). Measurements were made between 5.0° and 60.0° (2θ) with a scan rate of 2°/min.

#### 2.2.5. Differential scanning calorimetry (DSC)

A TA Q1000 differential scanning calorimeter (DSC) (TA Instruments, New Castle, Delaware) equipped with T-Zero® technology, RSC90 automated cooling system, auto sampler and calibrated with indium was used to measure phase transitions and thermal analysis, using conditions similar to those previously reported [22,23]. The samples were heated from at least 0.00 °C–250.00 °C at a scanning rate of 5.00 °C/min. All measurements were carried out in triplicate (n = 3).

#### 2.2.6. Hot stage microscopy (HSM) under cross-polarizers

Using conditions similar to previously reported [22,23], hot-stage microscopy (HSM) was performed using a Leica DMLP cross-polarized microscope (Wetzlar, Germany) equipped with a Mettler FP 80 central processor heating unit and Mettler FP82 hot stage (Columbus, OH). Samples were heated from at least 25.0 °C–250.0 °C at a heating rate of 5.00 °C/min.

#### 2.2.7. Karl Fisher titration (KFT)

Using conditions similar to previously reported [22,23], the residual water content of all SD powders were quantified analytically by coulometric Karl Fischer titration (KFT) with a TitroLine 7500 trace titrator (SI Analytics, Weilheim, Germany).

#### 2.2.8. Attenuated total reflectance (ATR)-fourier-transform infrared (FTIR) spectroscopy

ATR-FTIR spectra were obtained with a Nicolet Avatar 360 FTIR spectrometer (Varian Inc., California) equipped with a DTGS detector and a Harrick MNP-Pro (Pleasantville, New York) attenuated total reflectance (ATR) accessory. Each spectrum was collected for 32 scans at a spectral resolution of 2 cm^−1^ over the wavenumber range of 4000–400 cm^−1^. These conditions were similar to those previously reported [22, 23].

#### 2.2.9. In vitro aerosol dispersion performance

The aerosol dispersion performance of SD L-Car and SD L-Car HCl particles was tested using the Next Generation Impactor™ (NGI™) (MSP Corporation, Shoreview, MN) in accordance with USP Chapter <601> specifications on aerosols [25] and using conditions similar to previously reported [22,23]. Two FDA approved human DPI devices, HandiHaler® (Boehringer Ingelheim, Ingelheim, Germany) and NeoHaler™ (Novartis AG, Stein, Switzerland) were tested. All experiments were conducted at an airflow rate (Q) of 60 L/min adjusted and measured before each experiment using a Copley DFM 2000 digital flow meter (Copley Scientific, Nottingham, United Kingdom). The NGI™ was connected to a Copley HCP5 high-capacity vacuum pump through a Copley TPK 2000 critical flow controller. The mass of powder deposited on each stage was quantified by gravimetric method using type A/E glass fiber filters with diameter 55 mm (PALL Corporation, Port Washington, New York) and 75 mm (Advantec, Japan). Clear HPMC (hydroxyl-propylmethylcellulose) size 3 inhalation grade capsules (Quali-V, Qualicaps, North Carolina) were filled with approximately 10 mg of powder. Three capsules were used in each experiment. *In vitro* aerosolization was evaluated in triplicate (n = 3) under ambient conditions.

For the NGI, Q = 60 L/min, the D_a50_ aerodynamic cutoff diameter for each NGI stage was calibrated by the manufacturer and stated as: Stage 1 (8.06 μm); Stage 2 (4.46 μm); Stage 3 (2.82 μm); Stage 4 (1.66 μm); Stage 5 (0.94 μm); Stage 6 (0.55 μm); and Stage 7 (0.34 μm). The emitted dose (ED) based on the total dose (TD) used, the respirable fraction (RF) based on total deposited dose (DD) and the fine particle dose (FPD), the fine particle fraction (FPF) were calculated using the following equations:

Equation 1 $$Emitted\;Dose\;fraction(ED\text{\%})=\frac{ED}{TD}\times 100\text{\%}$$

Equation 2 $$Fine\;Particle\;Fraction(FPF\text{\%})=\frac{FPD}{ED}X100\text{\%}$$

Equation 3 $$Respirable\;Fraction(RF\text{\%})=\frac{FPD}{DD}\times 100\text{\%}$$

#### 2.2.10. In vitro cell dose response assay in a 2-D cell culture

The effects of the different SD formulations on cell proliferation were analyzed by measuring the response of lung adenocarcinoma and bronchoalveolar carcinoma cells (A549 and H358, respectively) to different concentrations using similar conditions as previously reported [23,24,26]. Both cell lines were grown in a growth medium including Dulbecco’s modified Eagle’s medium (DMEM), Advanced 1x, 10% (v/v) fetal bovine serum (FBS), Pen-Strep (100 U/mL penicillin, 100 μg/mL), Fungizone (0.5 μg/mL amphotericin B, 0.41 μg/mL sodium deoxycholate), and 2 mM L-Glutamine in a humidified incubator at 37 °C and 5% CO_2_.

A549 and H358 cells were seeded in 96-well plates at 5000 cells/well and 100 μL/well and were allowed 48 h to attach. The cells were then exposed to different concentrations of the SD formulations dissolved in DMEM. A volume of 100 μL of the drug solution was added to each well. Seventy-two (72) hours after exposure, 20 μL of 20 μM resazurin sodium salt were added to each well and incubated for 4 h. At this point, the fluorescence intensity of the resorufin (metabolite) produced by viable cells was detected at 544 nm (excitation) and 590 nm (emission) using the Synergy H1 Multi-Mode Reader (BioTek Instruments, Inc., Winooski, Vermont). The relative viability of cell line was calculated as follows with equation (4):

Equation 4 $$\text{Relative}\;\text{viability}(\text{\%})=\frac{\text{Sample}\;\text{fluorescence}\;\text{intensity}}{\text{Control}\;\text{fluorescence}\;\text{intensity}}\times 100\text{\%}$$

Analysis of Variance (ANOVA) statistical method was used to compare the relative viability between the treated *vs.* the non-treated cells. SigmaPlot 13 (SYSTAT Software, Inc, San Jose, CA) was employed for this purpose.

#### 2.2.11. In vitro transepithelial electrical resistance (TEER) analysis upon particle exposure to lung epithelial cells

Calu-3 lung epithelial cells were grown in a growth medium including Eagle’s minimum essential medium (EMEM), 10% (v/v) fetal bovine serum (FBS), Pen-Strep (100 U/mL penicillin, 100 μg/mL), Fungizone (0.5 μg/mL amphotericin B, 0.41 μg/mL sodium deoxy-cholate) in a humidified incubator at 37 °C and 5% CO_2_, as previously reported [24]. The cells were seeded onto 12-well Transwell inserts (Costar 3460, Corning, New York) from Fisher Scientific (Hampton, New Hampshire) at a density of 250,000 cells/well. They were allowed to attach and to form a monolayer at liquid-covered culture (LCC) conditions. Once the monolayer was formed, air-interface culture (AIC) was induced by removing the cell media from the apical side of the transwell inserts. The TEER values were measured using an EndOhm 12 mm Culture Cup (World Precision Instruments, Sarasota, Florida) and when they were around 500 Ω cm^2^, representative formulations of SD L-Car at a concentration of 1000 μM were added to the inserts. The liquid aerosol formulations were delivered to the Calu-3 cells at ALI using a Penn Century MicroSprayer® Aerosolizer Model IA-1B (Wyndmoor, PA) [26]. up to 7 days after aerosol treatment, as previously reported [24,27]. For TEER measurements, 0.5 mL of media was added to the apical side of the Transwells 5 min before measurement and then immediately removed to return the cells to ALI conditions.

Furthermore, TEER measurements were also performed on H441 cells in ALI conditions to test L-Car HCl formulations. H441 cell line was grown in T-75 culture flasks in an atmosphere of 5% CO_2_ at 37 °C. H441 cells were maintained in proliferation medium (RPMI 1640-Gibco) containing 10% fetal bovine serum (FBS), 1% PenStrep and 1% of GlutaMAX. Once they were confluent (90%), cells were seeded onto 12-well Transwell inserts® at a density of 250,000 cells/well in proliferation medium (0.5 mL in the apical and 1.5 mL in the basolateral chambers). The seeding day was defined as Day 0. Cells were allowed to attach before media was changed to polarization medium. The basal media was changed every other day until a monolayer was formed. Once that happened, cells were then fed with polarization medium, which was made up of base medium RPMI 1640 containing 4% FBS, 1% penicillin-streptomycin, 1% GlutaMAX, 1% insulin-transferrin-selenium, and 200 nM dexamethasone. Three days later, the polarization medium was removed from the apical compartment, leaving the apical surface of the cells exposed at the air-liquid interface (ALI). Medium was changed every two days. The maximum expected ALI TEER was around 250 Ω/cm^2^. Once they reached that value, cells were exposed to the drug formulations. TEER values were read after 3 h of treatment and every day to monitor the behavior of the monolayer. The culture procedure for H441 cells has been reported previously [28,29]. An EndOhm 12 mm Culture Cup (World Precision Instruments, Sarasota, Florida) was utilized to measure the transepithelial electrical resistance of the cells. For TEER measurements, 0.5 mL of media was added to the apical side of the Transwells 5 min before measurement and then immediately removed to return the cells to ALI conditions.

The statistical method used to compare the TEER values between the treated *vs.* the non-treated cells was Analysis of Variance (ANOVA). SigmaPlot (Systat Software, Inc) was employed for this purpose.

#### 2.2.12. In vitro cell dose response assay in a 3D human pulmonary primary cell culture

SmallAir™ (Epithelix, Geneva, Switzerland) 3D human pulmonary primary cells were reconstituted *in vitro* with corresponding SmallAir™ special growth media (Epithelix, Geneva, Switzerland) after received. After 3 days of incubation at 37 °C and 5% CO_2_, experiments were performed. The cells were exposed to 1000 μM of L-Car in both forms, separately, dissolved in growth media. After 72 h of incubation, the inserts were rinsed with a 6 μM Resazurin solution in order to get rid of the remaining red phenol from the cell growth media. The inserts were transferred to a new 24 well plate filled will 500 μL/well of Resazurin solution and then 200 μL/well was added in the apical surface. After 1 h of incubation, 100 μL from the apical side were transferred to a 96-black well plate. The fluorescence intensity was measured and calculated, as previously mentioned using Equation (4). This protocol provided by the vendor [30].

#### 2.2.13. In vitro transepithelial electrical resistance (TEER) analysis upon particle exposure to 3D human small airway epithelia

The cells were incubated as it was stated above for three days. Then, the cells were exposed to 1000 μM of L-Car in both forms dissolved in growth media. TEER values were measured using EVOMX Epithelial VoltohmMeter and STX2 electrode (World Precision Instruments, Sarasota, FL) before and after exposure to the drug solution. The response was measured after 3 h of exposure and every 24 h for 5 days. Every time the TEER measurement was obtained, the media was removed from the apical surface to leave the cells at ALI. This protocol was provided by the vendor [30].

#### 2.2.14. In vivo MCT-Rat model of PH

Twenty male 3-week old Sprague Dawley rats with a body weight in the range of 260g–315g upon receipt were purchased from Charles River Laboratories International Inc. (Wilmington, MA). Rats were housed in the University of Arizona Animal Care (UAC) Facility for at least 1 week for to acclimatize prior to the initiation of experiments. Animals were kept in a 12-h light/dark cycle at an ambient temperature of 22 °C and received standard rodent food and water ad libitum. All experimental procedures were approved by the Institutional Animal Care and Use Committee (IACUC) at The University of Arizona.

Monocrotaline (MCT)-induced PH in the rat is a validated preclinical model of PH [31–33]. Rats were injected with a single intraperitoneal (IP) injection of MCT (Sigma-Aldrich®, St. Louis, MO) at a dose of 60 mg/kg which results in development of PH 4-weeks after exposure. Four groups with 5 rats/group were defined for the study: a) control (Phosphate buffered saline, PBS); b) control (L-Car); c) MCT PBS; and d) MCT L-Car. After 14 days [33], targeted pulmonary inhalation aerosol delivery non-invasively was carried out using the Penn-Century MicroSprayer® Aerosolizer Model IA-1B. The dose was 200 mg/kg of L-Carnitine dissolved in PBS. The dose was delivered once-daily for 14 days under local short-acting anesthesia using inhalation of 1–5% isoflurane vapor in oxygen gas mixture. The rodent Microsprayer® atomizer was placed on the surface of the tongue of the rat and then slowly moved down into the larynx/trachea region located at the front of the esophagus/neck area to just above the first bifurcation of the bronchi (carina) directly visualized. A small-animal rodent laryngoscope (Penn-Century Inc. Wyndmoor, PA) was used to directly visualize the trachea and carina of the rats. The aerosols were sprayed into the lungs of the animals using the Medallion® glass syringe (Merit Medical Systems, South Jordan, Utah) containing the liquid and connected to the Penn-Century Microsprayer® atomizer and then was removed from the throat of the animal. Total aerosol treatment took ~5–7 min per rat per dose. Rats were closely monitored after recovery and weighed every week. On day 28 post-PH induction, animals were euthanized with a 100 mg/kg ketamine/10 mg/kg xylazine drug cocktail by IP injection:

As previously reported [33], the right ventricular systolic pressure (RVSP) was measured in all groups using a pressure transducer catheter customized for the rat and inserted inside right internal jugular vein. Rats were euthanized and the heart dissected for measurement of Fulton Index (RV/LV + septum). All hemodynamic variables were measured continuously utilizing the Gould Ponemah Physiology Platform (Version 4.2) and Acquisition Interface (Model ACG-16, Gould Inc., Cleveland, OH), and recorded with a Dell Inspiron 5160 computer (Dell Inc., Round Rock, TX) [7]. Animals were euthanized and a thoracic incision was made to harvest the lungs, collect terminal blood by cardiac puncture, and collect the bronchioalveolar lavage fluid (BALF).

#### 2.2.15. L-carnitine quantification assay

The amount of L-Carnitine in plasma samples was quantified using the Abcam quantification assay kit (ab83392, Abcam, Cambridge, MA). Plasma samples were deproteinized following the kit protocol. The amount of L-Carnitine was then calculated by fluorescence using a Synergy H1 Multi-Mode Reader (BioTek Instruments, Inc., Winooski, VT) at Ex/Em = 535/587 nm.

#### 2.2.16. Statistical analysis

Design-of-experiments (DoEs) was conducted using Design-Expert® 8.0.7.1 software (Stat-Ease Corporation, Minneapolis, MN). A multi-factorial design for the SD powders was utilized for *in vitro* aerosol testing. Interaction of the inhaler device resistance and the spray drying parameters were evaluated. In addition, the *in vitro* aerosol performance between the two different forms of L-Car was compared using Analysis-of-Variance (ANOVA) test using Design-Expert®. The different interactions on the performance of the formulations were evaluated using the 3-D surface plot generated from Design-Expert®. All *in vitro* experiments were performed in at least triplicate (n = 3) except where indicated. Results are expressed as mean ± standard deviation.

## 3. Results

### 3.1. Scanning electron microscopy (SEM)

**Fig. 2.**
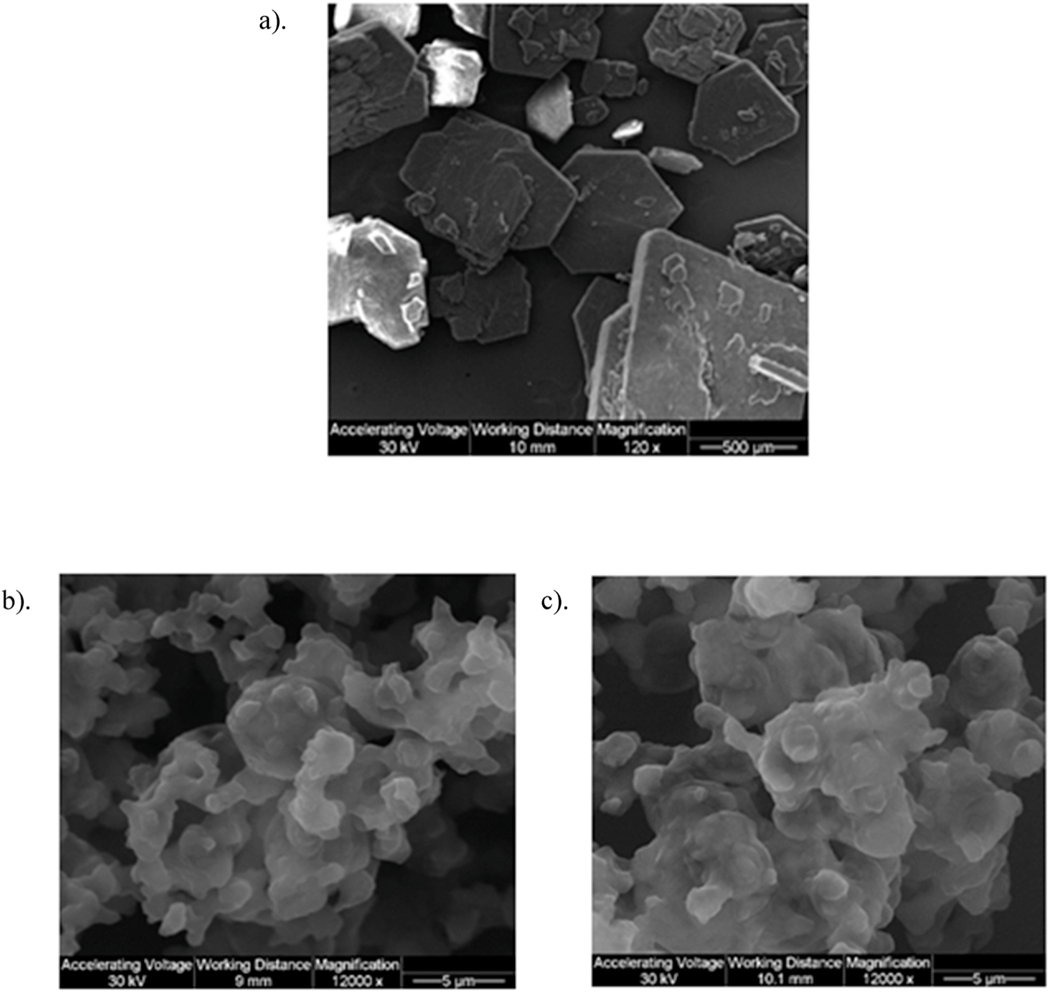
SEM micrographs of: a). Raw L-Car; b). SD L-Car (25% PR); and c). SD L-Car (50% PR).

**Fig. 3.**
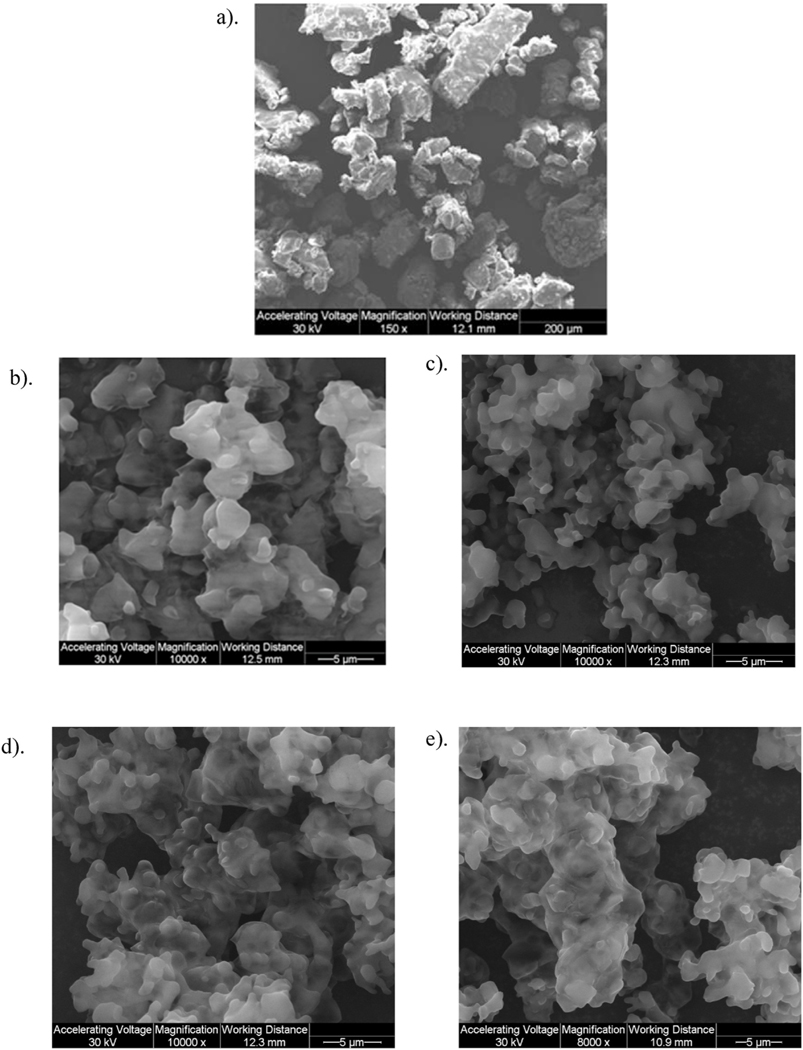
SEM micrographs of: a). Raw L-Car HCl; b). SD L-Car HCl (25% PR); c). SD L-Car HCl (50% PR); d). SD L-Car HCl (75% PR); and e). SD L-Car HCl (100% PR).

Size and morphology of Raw and SD particles were visualized by SEM, as shown in Figs. 2 and 3. SD L-Car was successfully produced at 25% PR and 50% PR, although other pump rates were tried with no resulting powder On the contrary, SD L-Car.HCl successfully formed powders at 25% PR, 50% PR, 75% PR, and 100% PR. Both forms of carnitine showed a significant reduction in size and change in morphology from the raw to the SD powders. All SD powders exhibited sintering and some aggregation as nanoaggregates. The aggregates in all SD powders displayed uniform geometry.

### 3.2. Particle sizing and size distribution by image analysis of SEM micrographs

As shown in Table 3, SD powders of L-Car (25% PR and 50% PR) had a broad distribution but the mean of the particle size was ~15.2 μm and 12.6 μm, respectively. SD systems of L-Car.HCl did not show such broad distributions and the mean size was ~5 μm. The large distributions obtained were due to the difficulty in analyzing the particle sizes of the different systems due to the aggregates that they were forming.

### 3.3. X-ray powder diffraction (XRPD)

**Fig. 4.**
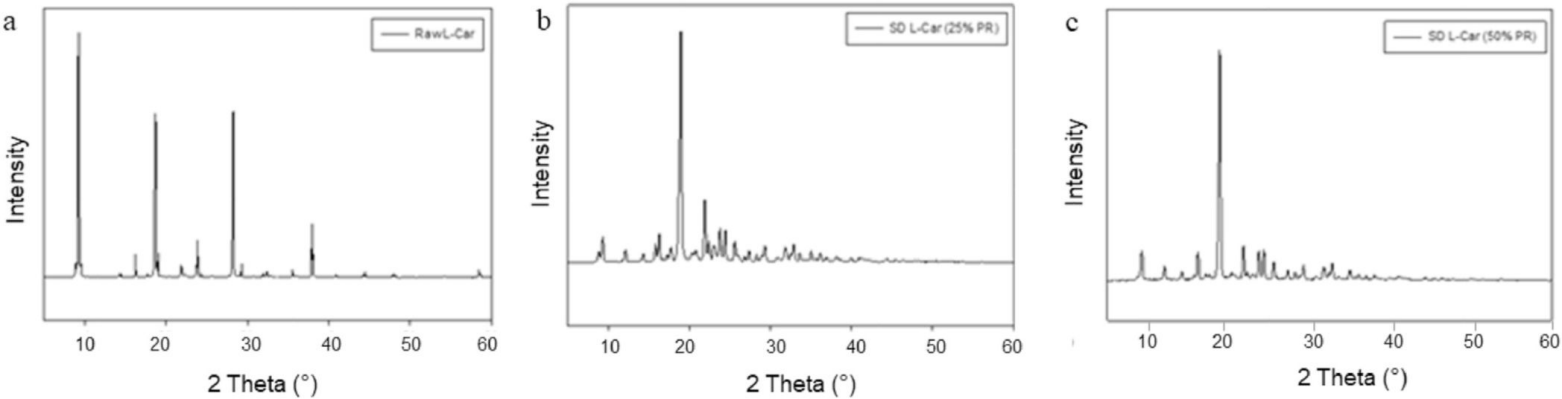
XRPD diffractograms of: a). Raw L-Car; b). SD L-Car (25% PR); and c). SD L-Car (50% PR).

**Fig. 5.**
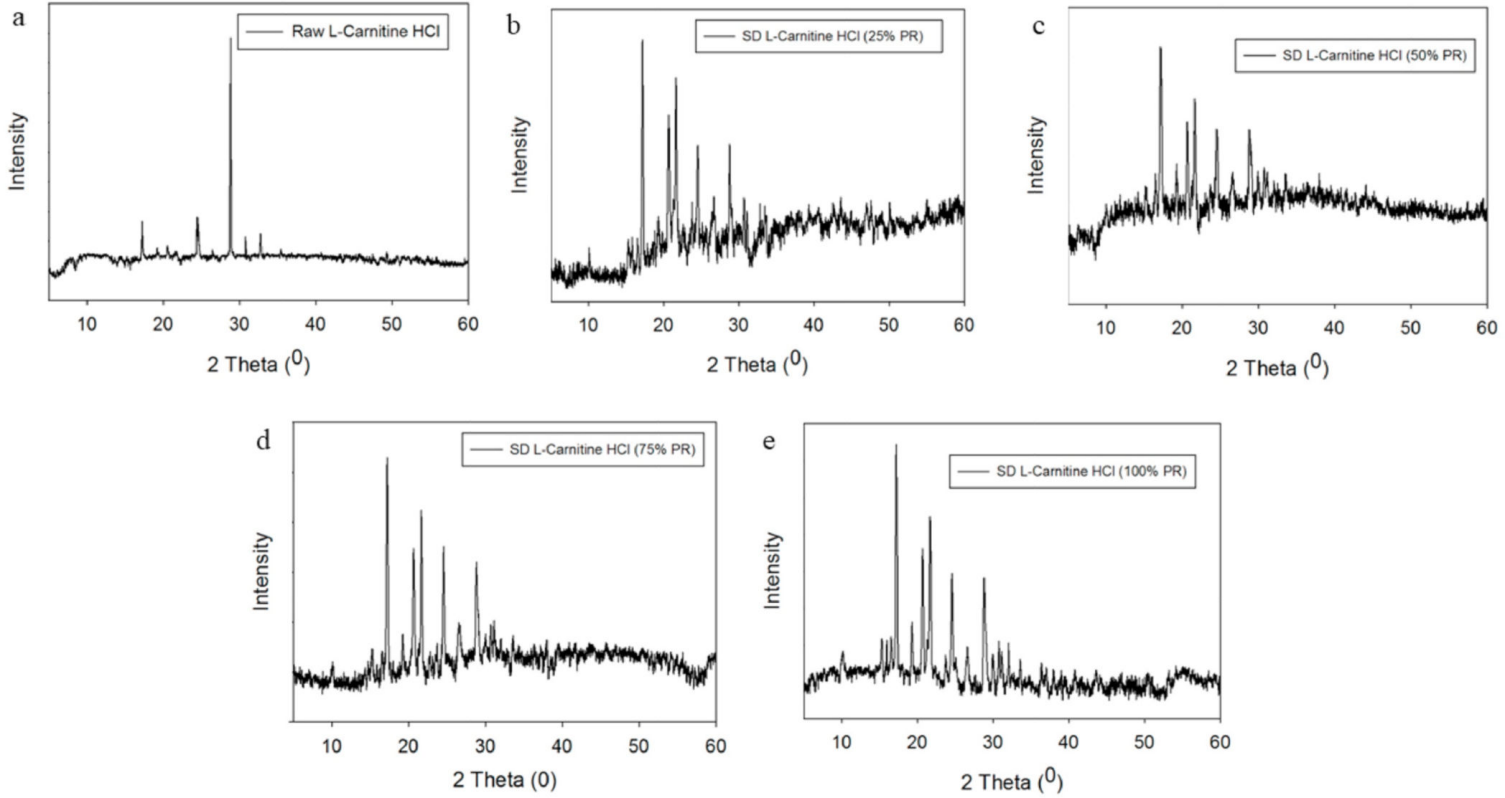
XRPD diffractograms of: a). Raw L-Car HCl; b). SD L-Car HCl (25% PR); c). SD L-Car HCl (50% PR); d). SD L-Car HCl (75% PR); and e). SD L-Car HCl (100% PR).

The XRPD pattern of raw L-Car, raw L-Car HCl and all SD powders showed sharp and intense peaks (i.e. long-range molecular order). Even though both L-Car forms were crystalline in the XRPD diffractometer, they were showing different diffraction patterns. As shown in Fig. 4, L-Car showed numerous distinctive peaks at precise diffraction angles of 2θ-degrees: specifically at 9.2, 16.2, 18.7, 23.9, 28.2 and 37.9 2θ-degrees, in agreement with the literature [34]. In contrast to L-Car (Fig. 4), L-Car.HCl (Fig. 5) showed distinctive peaks at different precise diffraction angles of 2θ-degrees: specifically at 17.2, 19.2, 24.4, 28.8, and 30.7 2θ-degrees.

### 3.4. Differential scanning calorimetry (DSC)

**Fig. 6.**
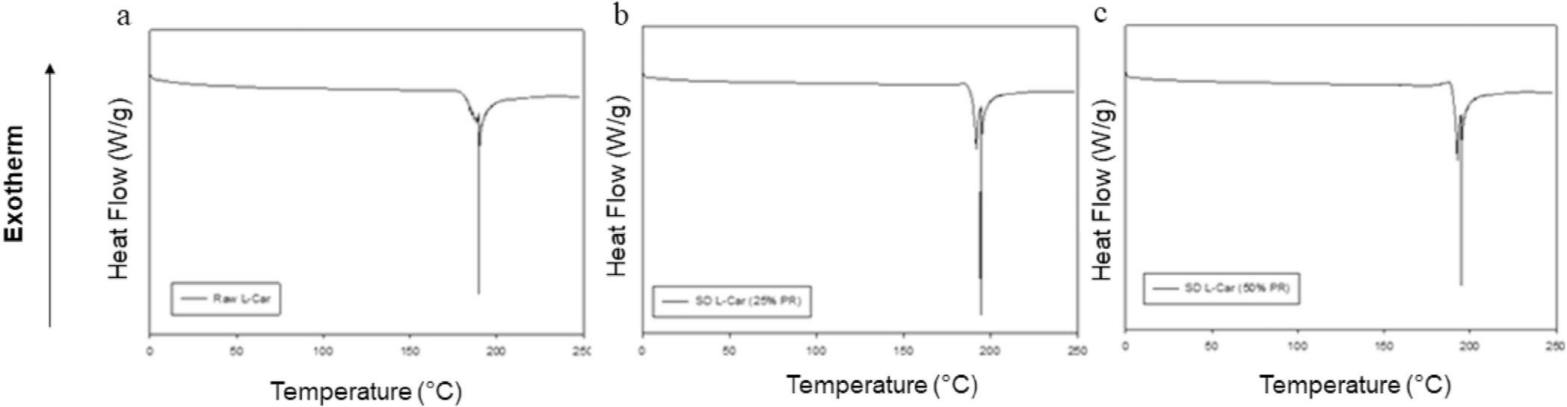
DSC thermograms at 5.00 °C/min heating rate under hermetically sealed conditions for: a). Raw L-Car; b). SD L-Car (25% PR); and c). SD L-Car (50% PR).

**Fig. 7.**
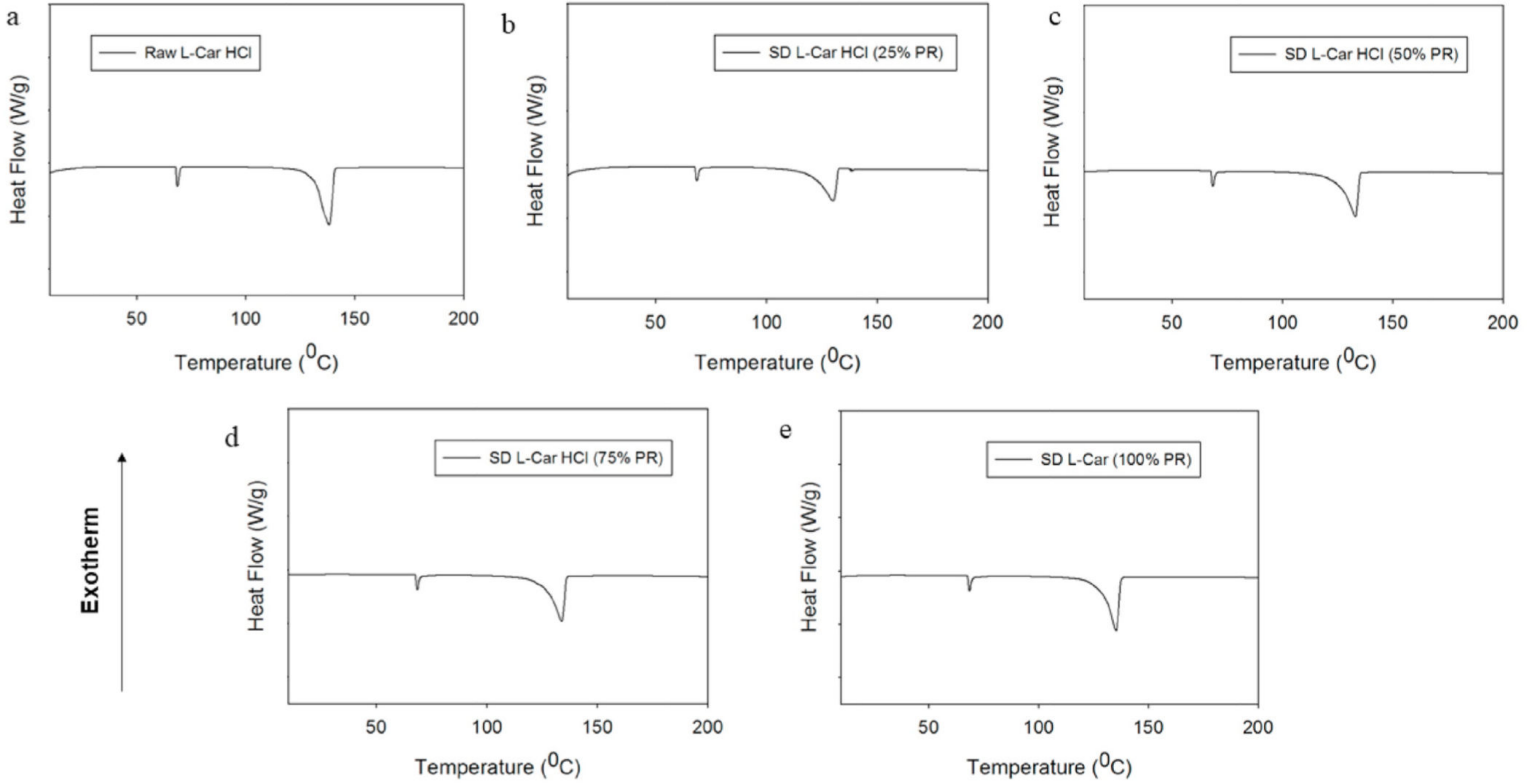
DSC thermograms at 5.00 °C/min heating rate under hermetically sealed conditions for: a). Raw L-Car HCl; b). SD L-Car HCl (25% PR); c). SD L-Car HCl (50% PR); d). SD L-Car HCl (75% PR); and e). SD L-Car HCl (100% PR).

The thermograms of the raw and SD powders are shown in Figs. 6 and 7. Raw and SD (25% PR and 50% PR) L-Car thermograms, showed a single endothermic transition at ~190 °C, which was in good agreement with the literature [35]. Raw L-Car.HCl and the corresponding SD powders showed two endothermic transitions. The first minor transition was at 68 °C, and the second major endothermic transition around 138 °C, which corresponded to the melting of the compound. This was in agreement with the literature [36]. Phase transition temperatures and enthalpies for all systems are listed in Tables 4 and 5.

### 3.5. Karl Fisher titration (KFT)

The residual water content values of all raw and SD powders were quantified analytically by coulometric KFT. Residual water content of all systems is presented in Table 6. Both raw L-Car and L-Car HCl and their corresponding SD systems exhibited low residual water content. L-Car and SD L-Car systems had mean values ranging between 2.78 and 3.02 % w/w, whereas L-Car.HCl and SD L-Car.HCl had mean values ranging between 0.45 and 2.69 %w/w indicating a drier product following advanced spray drying in closed-mode using non-aqueous feed solvent (methanol).

### 3.6. HSM under cross-polarizer lens

**Fig. 8.**
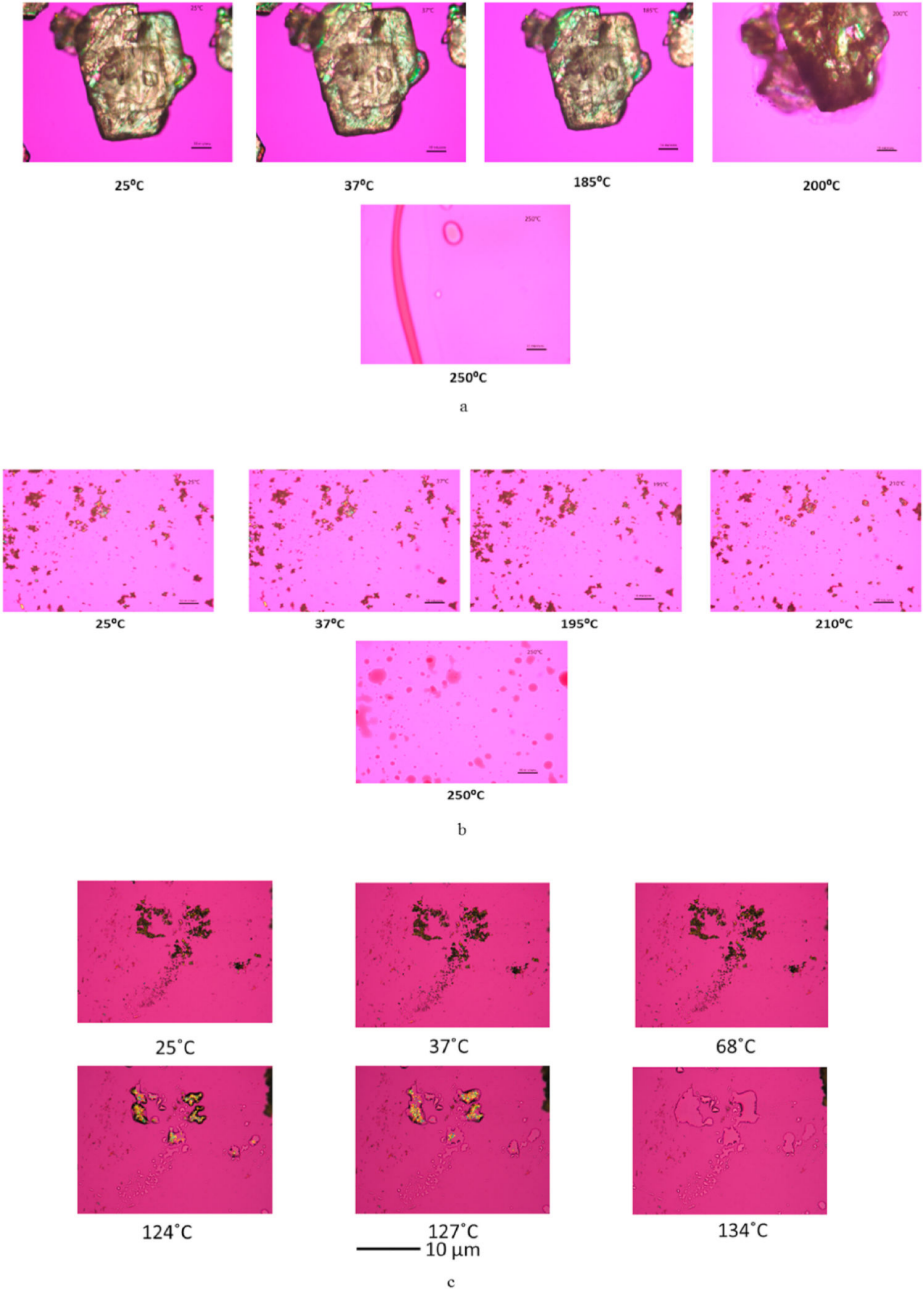
Representative HSM micrographs at various temperatures of: (a). Raw L-Car; (b).SD L-Car (50% PR); and (c). SD L-Car HCl (50% PR). Scale bar = 10 μm.

Representative images from HSM are shown in Fig. 8. Raw and SD L-Car exhibited birefringence under cross-polarizer lens. Raw and SD L-Car started melting at ~195 °C and completed melting at ~205 °C, in agreement with the literature [35]. Raw and SD L-Car.HCl also showed birefringence. There was only one evident phase transition at 135 °C, which corresponded to the complete melting of the powders and with literature as mentioned above [36].

### 3.7. Attenuated total reflectance-fourier-transform infrared (ATR-FTIR) spectroscopy

**Fig. 9.**
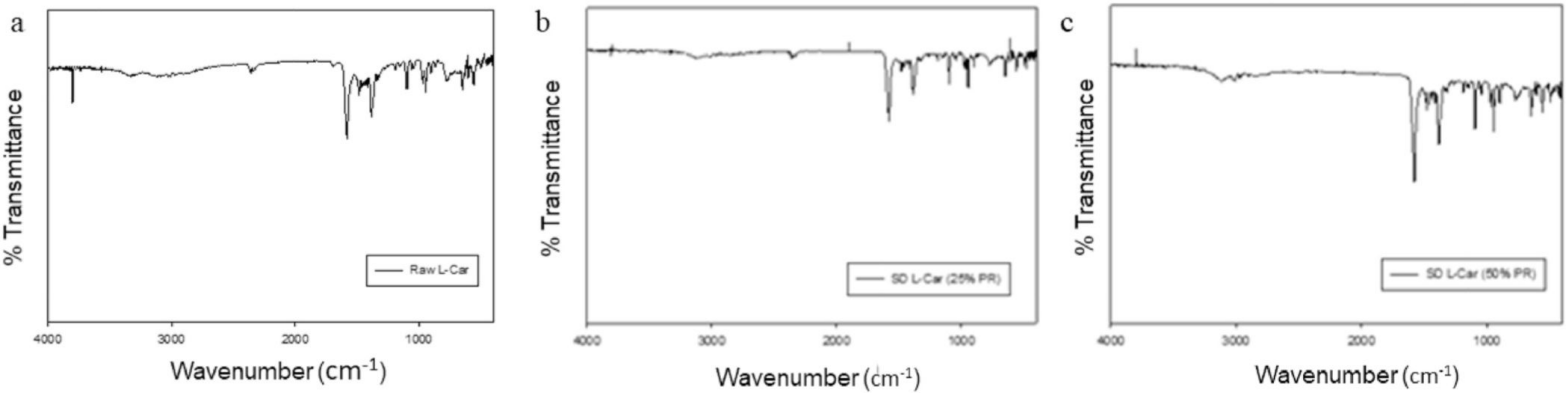
ATR-FTIR spectra of: a). Raw L-Car; b). SD L-Car (25% PR); and c). SD L-Car (50% PR).

**Fig. 10.**
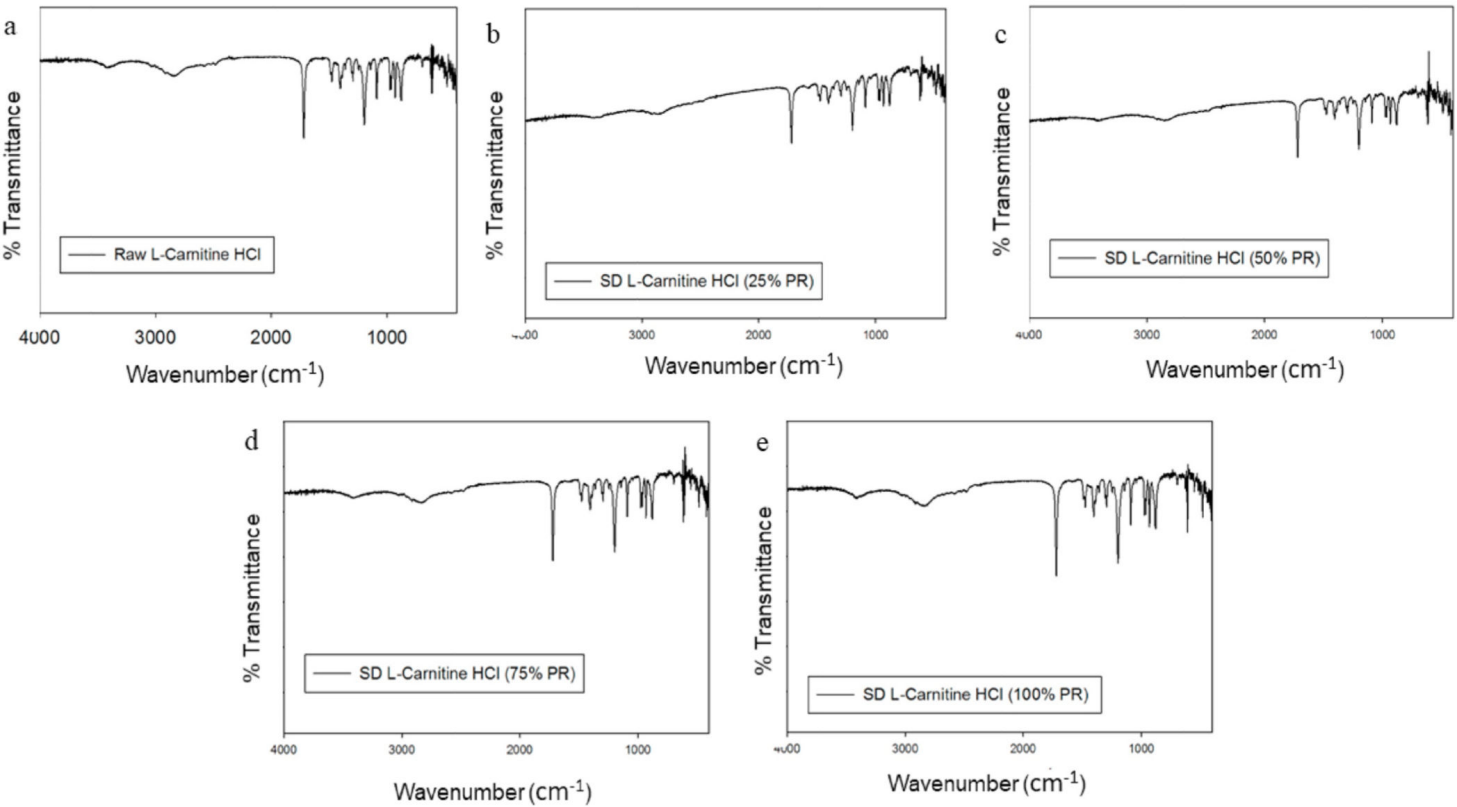
ATR-FTIR spectra of: a). Raw L-Car HCl; b). SD L-Car HCl (25% PR); c). SD L-Car HCl (50% PR); d). SD L-Car HCl (75% PR; and e). SD L-Car HCl (100% PR).

Spray dried particles and their raw counterparts underwent ATR-FTIR analysis to define the functional groups present in the systems, as shown in Figs. 9 and 10. ATR-FTIR of raw and SD of both forms of L- Car spectra were in good agreement with the literature, showing characteristics peaks at 1580, 1483, 1411, 1383, 968, 946 and 774 cm—1. The most characteristic band of L-Carnitine in both forms was in agreement with the literature [37].

### 3.8. In vitro aerosol dispersion performance

The aerosol dispersion parameters for SD L-Car and L-Car HCl systems are listed in Table 7. The ED of SD L-Car with the NeoHaler™ was better than with the HandiHaler®. The PR effect was also observed. In general, the ED was better at 25% PR using the NeoHaler™. In terms of the FPF, the percentage was very low using both devices, however, it was better at 50% PR and using the HandiHaler® device. In regard to the RF, there was a clear difference between the HandiHaler® and the NeoHaler™. The RF% was higher using the HandiHaler® at 50% PR. The MMAD values were high for all systems, except for SD-L-Car 50% PR using the HandiHaler® device. This was consistent with image analysis observations.

**Fig. 11.**
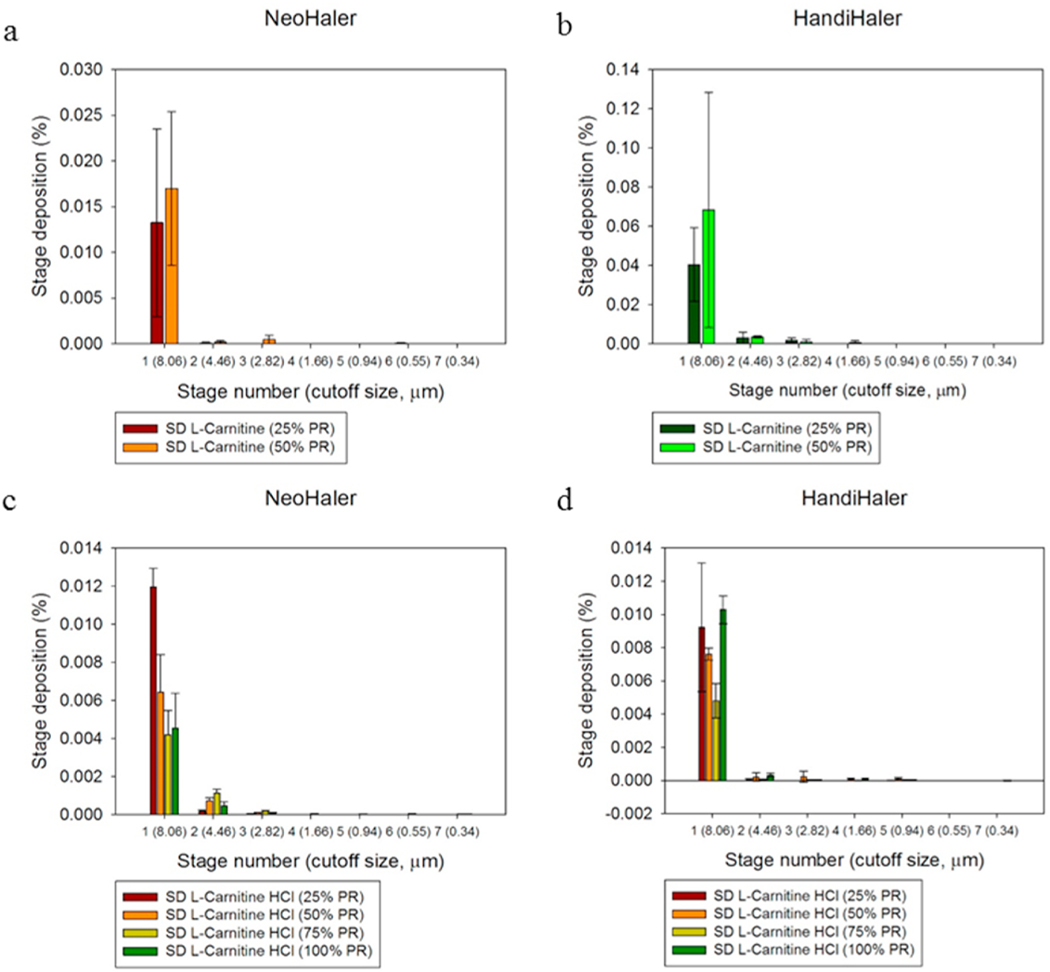
In vitro aerosol dispersion performance of: a). SD L-Car using the NeoHaler™ device; b). SD L-Car using the HandiHaler® device; c). SD L-Car.HCl using the NeoHaler™ device; and d). SD L-Car.HCl using the HandiHaler® device. (n = 3, Mean ± SD).

On the other hand, the ED of SD L-Car.HCl systems were all better using the NeoHaler™ device. With this device, the ED was better at 50% and 75% PR. Using the HandiHaler® device, the best ED was at 100% PR. The FPF values, were low as well, but they were comparable between the NeoHaler™ and the HandiHaler® and also at different PR. In terms of RF, there was a clear difference between the percentages of RF using the Neohaler™ than using the HandiHaler®. The highest RF was at 75% PR using the NeoHaler™. The MMAD values for the SD L-Car.HCl systems were highly scattered. From the table, it was established that the smallest MMAD values were obtained using the NeoHaler™ device at 75% PR. The mass deposition of all SD systems is shown in Fig. 11.

### 3.9. In vitro cell dose response assay

**Fig. 12.**
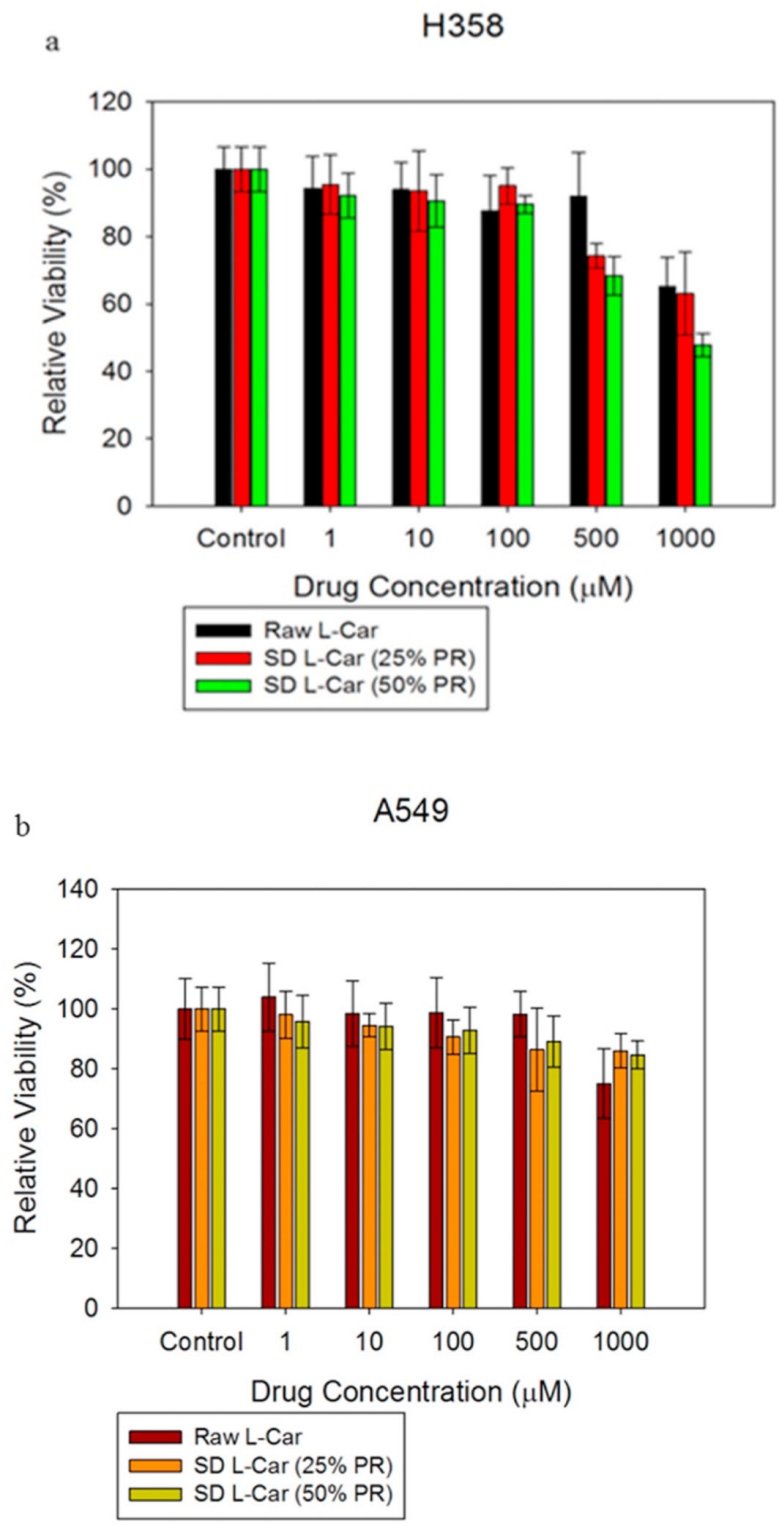
In vitro cell viability vs. drug concentration plots for human pulmonary cell lines: a). H358 and b). A549 cells after 72 h of exposure to different concentrations of L-Car. (n = 6, Mean ± SD).

**Fig. 13.**
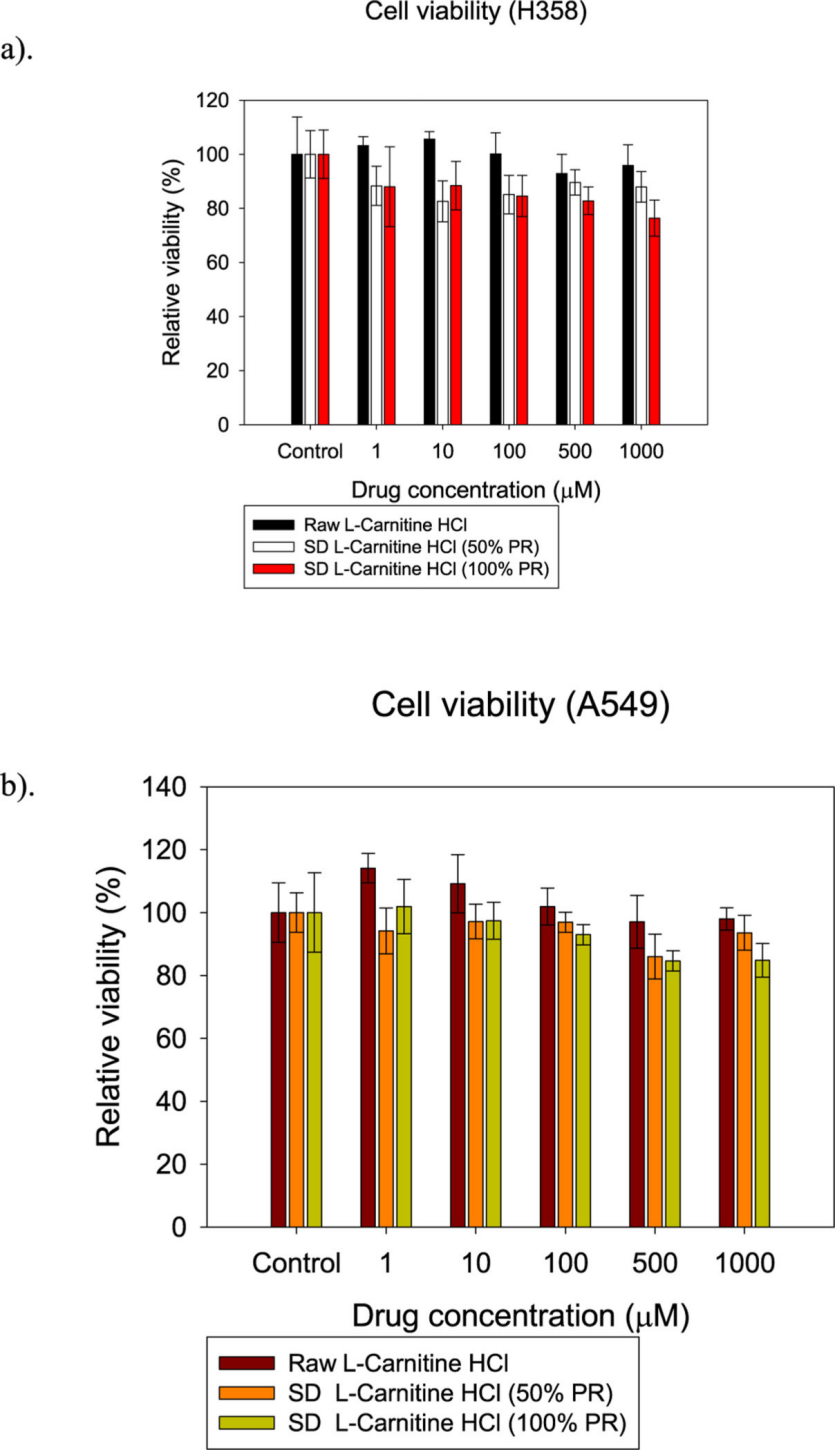
In vitro cell viability vs. drug concentration plots for human pulmonary cell lines: a). H358 and b). A549 cells after 72 h of exposure to different concentrations of L-Car HCl. (n = 6, Mean ± SD).

H358 and A549 cells were exposed to different concentrations of L-Car and L-Car HCL systems in order to test the dose response. Figs. 12 and 13 showed the dose-response of H358 and A549 cells after 72 h of exposure to different formulations of SD L-Car and SD L-Car.HCl. Most all formulations tested were shown to be safe at concentrations of 1 μM, 10 μM, and 100 μM. There was a slight decrease in viability when the cells were exposed to the higher concentrations of 500 μM and 1000 μM. There was not a statistically significant difference between the non-treated cells and the cells treated with 1 μM, 10 μM, and 100 μM in both cell lines (p-values >0.05). However, at concentrations of 500 μM and 1000 μM, the relative viability of the cells decreased, presenting a statistically significant difference between the relative viability of the control cells (no treatment) and the relative viability of the cells exposed to the different formulations (p-values < 0.05).

### 3.10. In vitro transepithelial electrical resistance (TEER) analysis upon particle exposure to lung epithelial cells

**Fig. 14.**
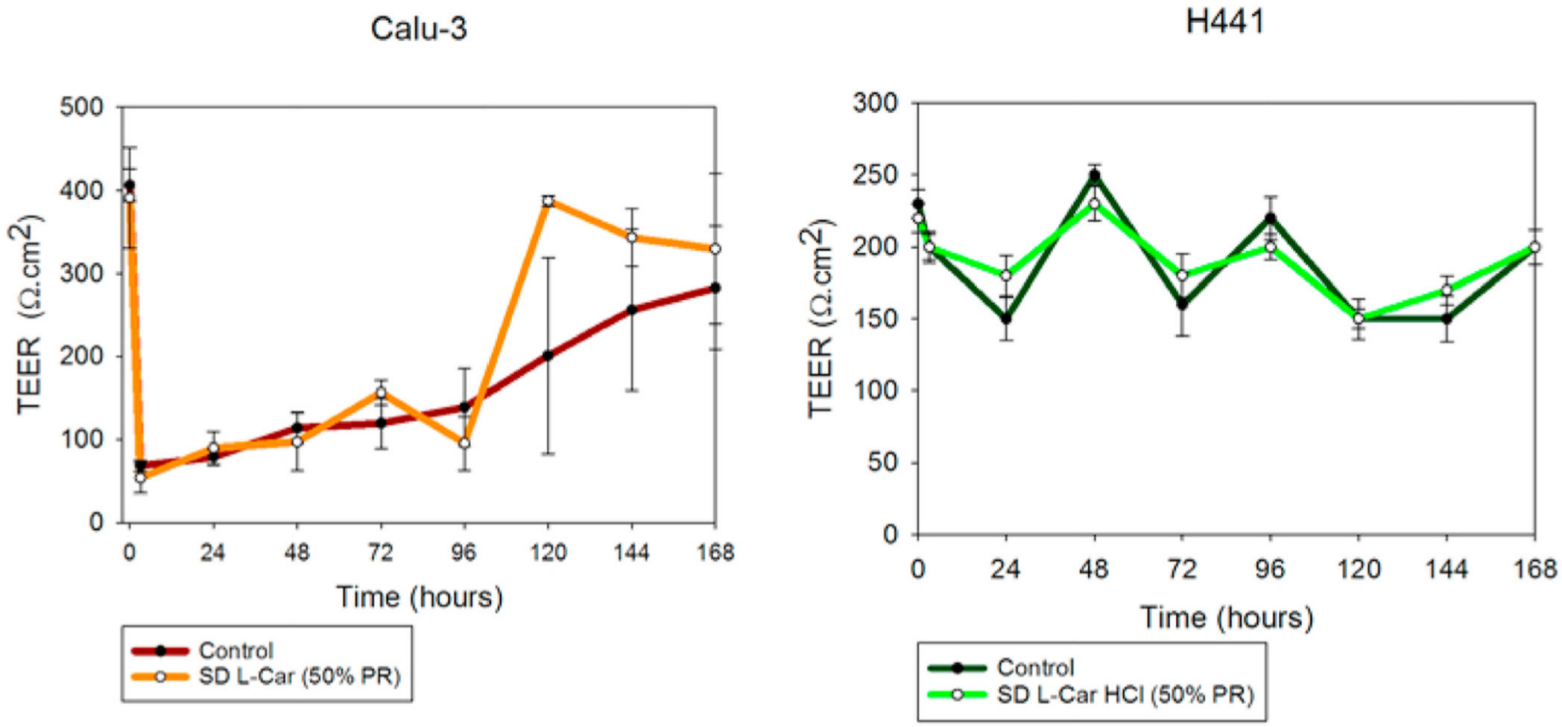
In vitro transepithelial electrical resistance (TEER) analysis of: a). Calu-3 and b). H441 lung epithelial cells exposed to 1000 μM SD L-Car and SD L-Car.HCl in AIC conditions. Calu-3 cells were exposed to the formulations using the Penn-Century MicroSprayer® Aerosolizer, whereas H441 cells were exposed to the formulations using a micropipette. (n = 3, mean ± SD).

TEER measurements were successfully performed on Calu-3 cells in ALI conditions to determine the effect of L-Car on the cell monolayer. The existence of a complete monolayer was confirmed by TEER values of approximately 500 Ω cm^2^ after seven days of exposure and by the observance of the monolayer via light microscopy (data not shown). As seen in Fig. 14, after 3 h of exposure, TEER values dropped significantly; however, after seven days of culturing, TEER values were ~500 Ω cm^2^. Moreover, a statistically significant difference was not observed between the TEER values before drug exposure and after seven days of cell culturing for each of the formulations (p-values ≥ 0.05).

For H441 cell monolayer, it can be observed in Fig. 14 (right) that there was a decrease in the TEER values after 24 h of exposure to L-Car HCl solutions. After 7 days, TEER values were almost the same as they were before with no statistically significant difference (p-values ≥ 0.05).

### 3.11. In vitro cell dose response assay in a 3-D cell culture

**Fig. 15.**
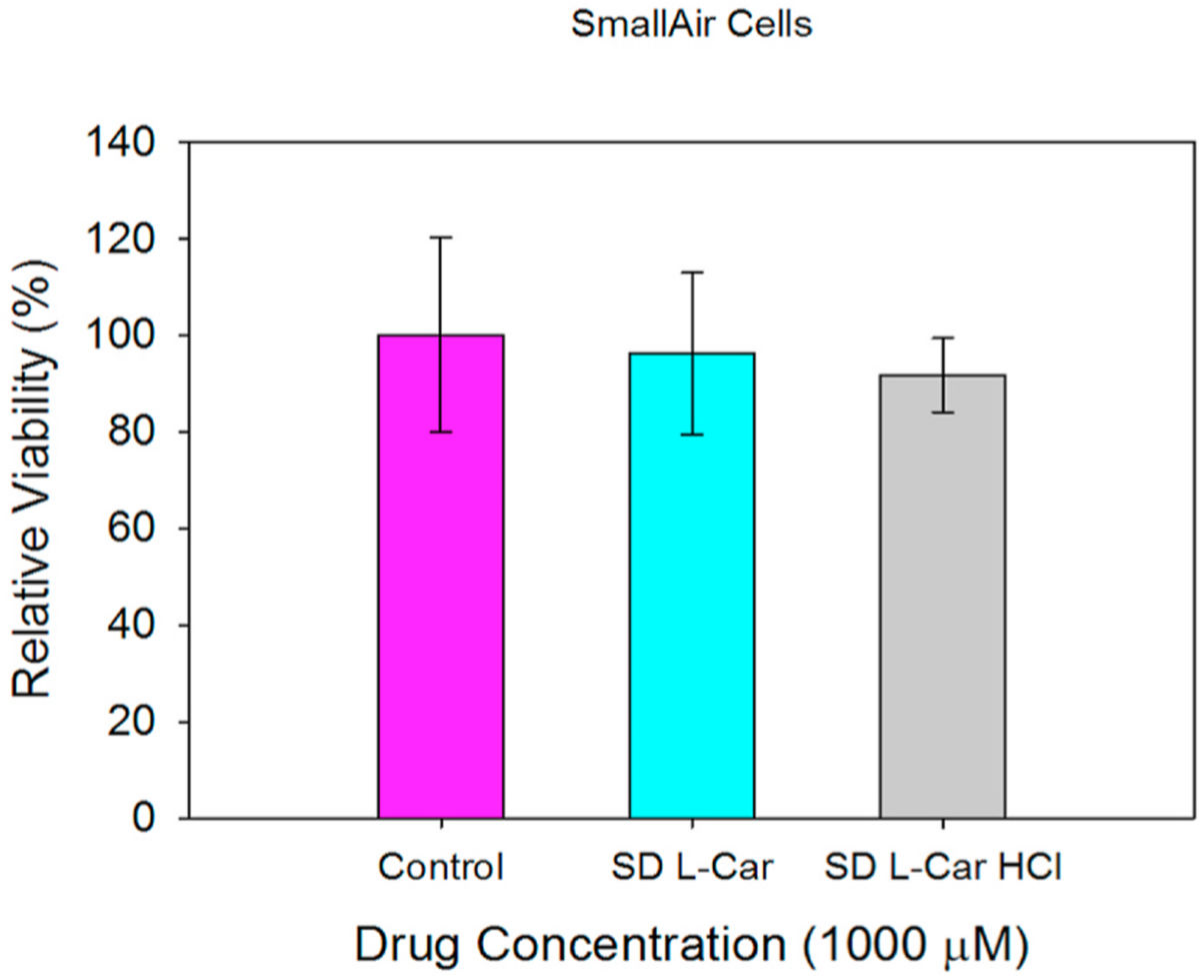
In vitro cell viability vs. drug concentration plots for SmallAir™ 3D human primary pulmonary cells after 72 h of exposure to different concentrations of SD L-Car and SD L-Car HCl. (n = 3, Mean ± SD).

After exposing the SmallAir™ cells to 1000 μM solutions of SD L-Car and SD L-Car.HCl, there was no change in the viability of the cells after 72 h of exposure. This is observed in Fig. 15.

### 3.12. In vitro transepithelial electrical resistance (TEER) analysis upon particle exposure to 3-D human small airway epithelia

**Fig. 16.**
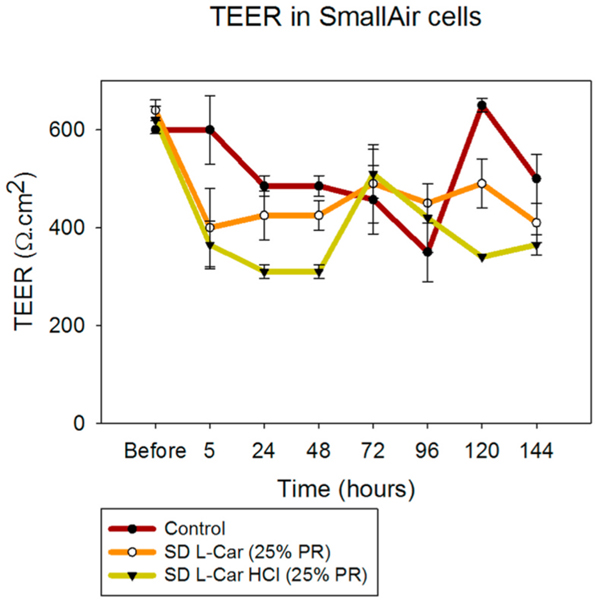
Transepithelial electrical resistance (TEER) analysis of SmallAir® 3D human primary pulmonary cells exposed to 1000 μM of SD L-Car and SD L-Car HCl in AIC conditions using a micropipette (n = 3, mean ± SD).

SmallAir™ cells were exposed to 1000 μM solutions of SD L-Car and SD L-Car HCl and TEER values were measured as described above. TEER values decreased after the exposure of the cells to the drug solutions. However, within a short time the TEER values increased and showed a healthy epithelium according to the vendor’s criteria (TEER values below 100 Ω/cm^2^_=_ non-healthy epithelium. TEER values above 100 Ω/cm^2^_=_ healthy epithelium) [30]. This is shown in Fig. 16.

### 3.13. In vivo MCT-Rat model of PH

**Fig. 17.**
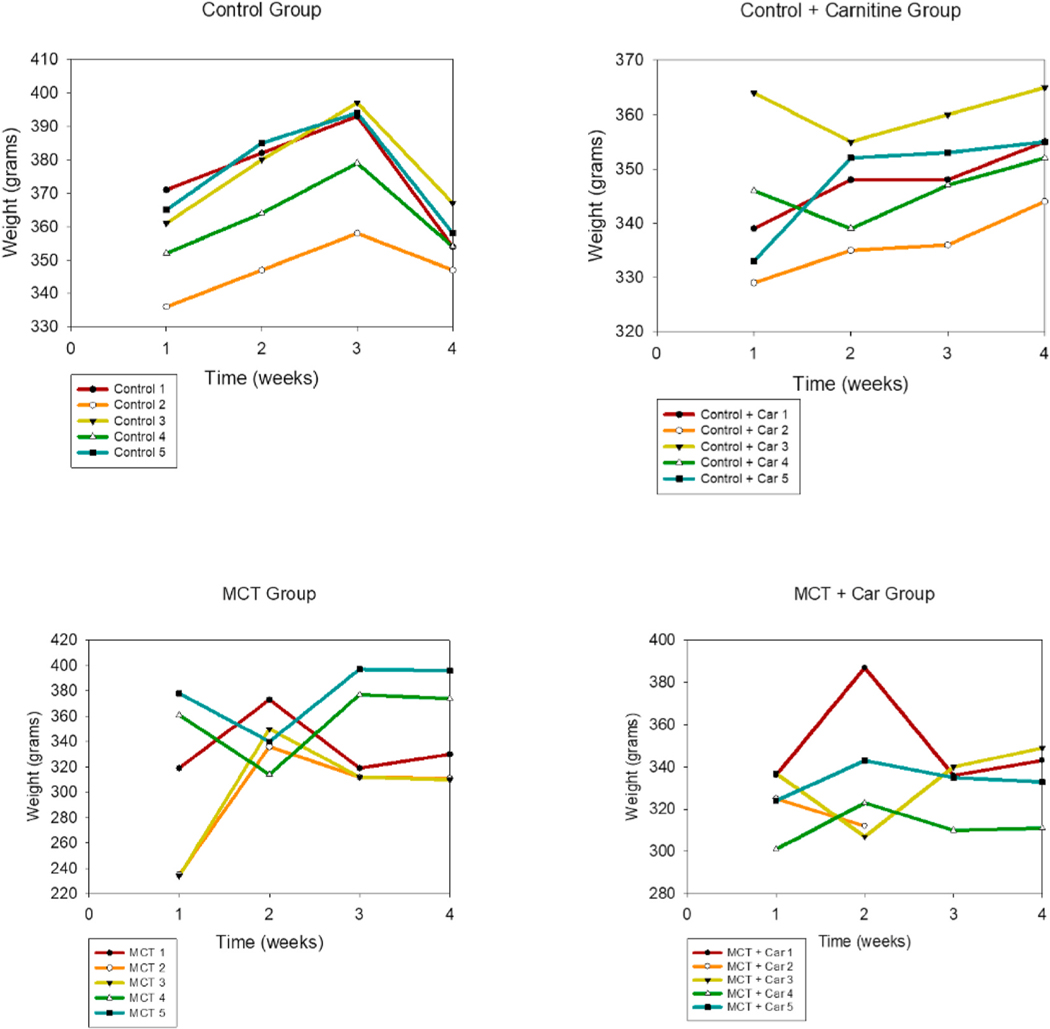
Weight vs. time plots for the male Sprague Dawley rat groups.

As observed in the weight *vs.* time plots (Fig. 17), the control and control-carnitine rats gained weight weekly. The MCT + PBS rats weight loss. The MCT + L-Car rats showed less weight loss and maintenance of weight.

**Fig. 18.**
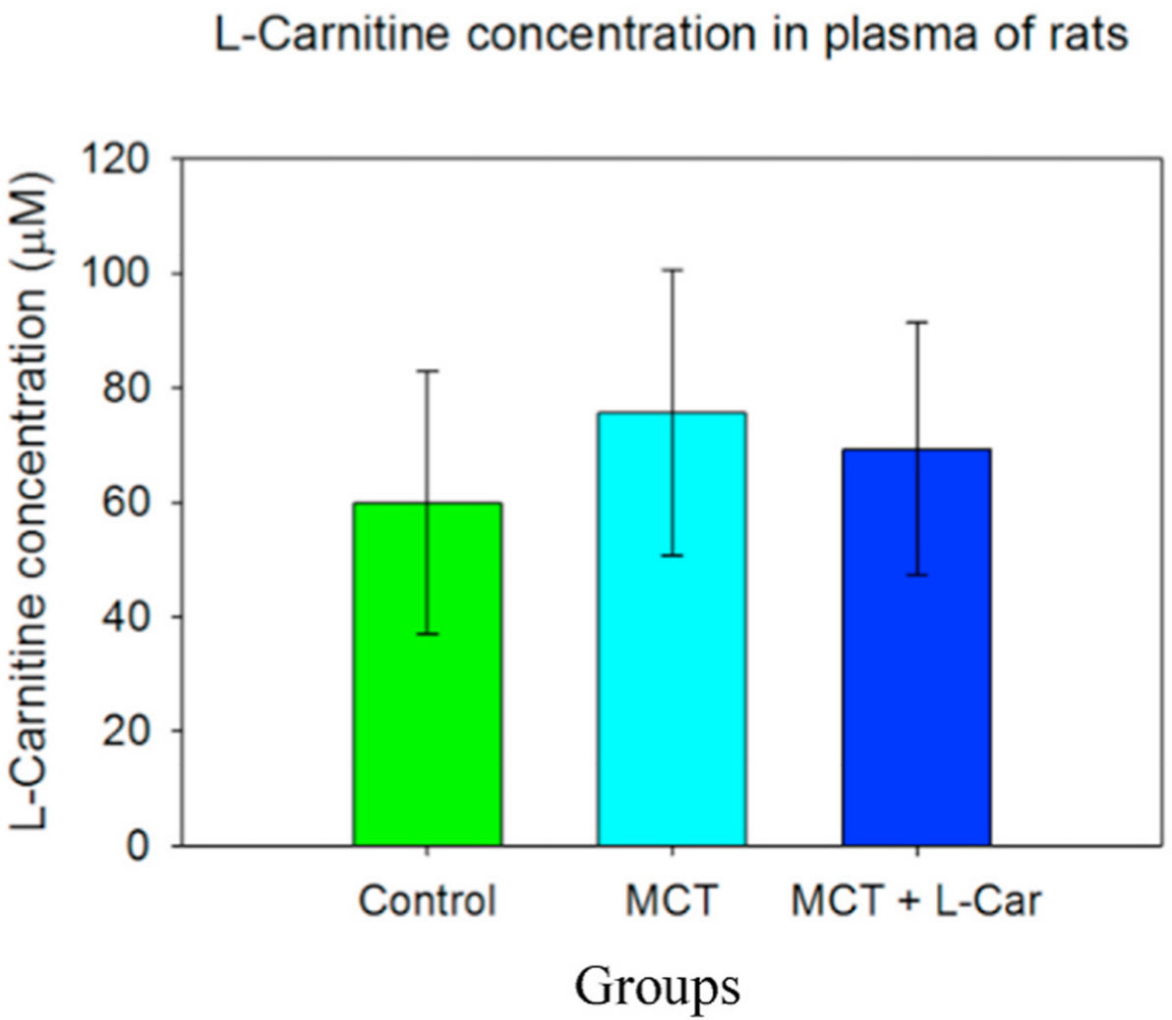
L-Car plasma concentrations in male Sprague Dawley rats. (n = 5, Mean ± SD).

There was also no statistically significant difference between the three groups in terms of the amount of L-carnitine in the analyzed plasma samples, as shown in Fig. 18 (p = 0.526).

**Fig. 19.**
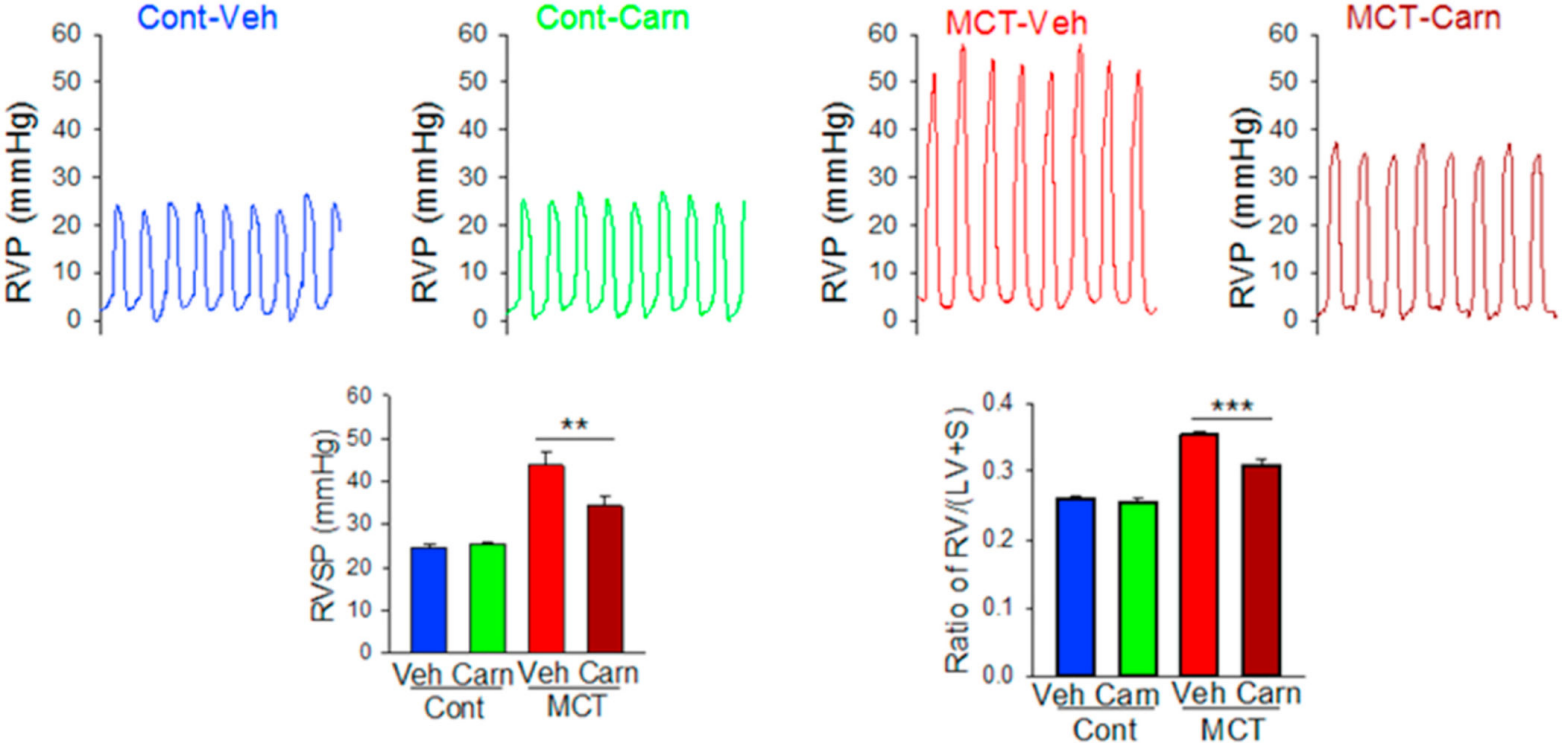
In vivo pulmonary hemodynamic data of L-Car in the MCT rat model of PH in male Sprague Dawley rats (n = 5, Mean ± SD).

As shown in Fig. 19, the control rats showed a right ventricular pressure (RVP) of ~25 mmHg (normal RVP in rats). The MCT + PBS rats showed a much higher RVP of ~45 mmHg. The MCT + L-Car rats showed a decreased RVP in comparison with the MCT rats of ~34 mmHg. There was mortality during the study, so the number of rats were differs between the beginning and end of the study. The MCT + L-Car group demonstrated significant reductions in RVSP (Table 8) when compared with MCT-only rats (RVSP 34.28 ± 2.34 mmHg *vs*. 43.70 ± 2.93 mmHg, p < 0.05). Similarly, MCT + L-Car rats had a significant reduction in Fulton Index (Table 8) when compared to the MCT group (0.31 ± 0.01 *vs*. 0.35 ± 0.01, p < 0.05).

## 4. Discussion

To the authors’ knowledge, this is the first time L-Carnitine has been formulated as a dry powder inhaler by spray drying in closed-mode. The co-factor L-Car exists in different forms. In this study, two forms were evaluated to determine which performed better as a DPI. Our data show that there were differences between formulations when comprehensive physicochemical characterization was evaluated. Only the 0.5% w/v resulted in powders after testing various concentrations. Different inlet temperatures were also tested and an inlet temperature of 150 °C was identified as optimal for L-Car and L-Car HCl. Four spray pump rates were tested to cover the spray drying pump rate (PR) range of 0%– 100%. Specifically, 25% (low), 50% (medium), 75% (medium-high), and 100% (high) PR values were systematically tested. Only the low PR of 25% and medium PR of 50% was successful for L-Car, whereas all four PR conditions (i.e. low, medium, medium-high, and high) covering the entire spray drying pump rate range were successful for L-Car.HCl. These data emphasize the effect that the salt vs. non-salt form of a drug can have on the spray drying process in producing powders. Condensation on the cyclone and on the collector vessel was observed at 75% PR (medium-high) and 100% PR (high) when L-Car was spray dried. This indicated there was insufficient drying time allowed during the primary and secondary drying processes in the spray dryer system for complete evaporation of methanol and the formation of dry particles at the medium-high (75%) and high pump (100%) rates. However, the morphology of the particles was similar for all the systems. The particles showed sintering, aggregation, wrinkled surfaces and it was not easy to distinguish single particles. Nanostructures were visible on the surface of the particles leading to their aggregation. This occurrence made difficult to size the particles using SigmaScanPro™. Aggregation of particles gave large particle size and broad distributions in some systems.

The XRPD diffraction patterns (Figs. 4 and 5) confirmed that both L-Car and L-Car.HCl possessed a long-range molecular order as indicated by the sharp and intense peaks of all the diffractograms. The XRPD diffraction patterns of SD L-Car and SD L-Car.HCl powders were characteristic of crystals, clearly indicating that the SD powders remained in the crystalline state after the spray drying process but crystalline polymorphs could have resulted following spray drying to enable the retention of crystallinity following the advanced spray drying process under these reported conditions. The DSC thermograms (Figs. 6 and 7) confirmed the crystalline form of raw L-Car, raw L-Car.HCl, and their corresponding spray dried powders when showing a main endothermic phase transition and the absence of a glass transition temperature (T_g_) i. e. absence of an amorphous phase within the detection limits of the DSC. The main endothermic phase transition of both L-Car and L-Car HCl corresponded with the literature as the melting point (T_m_) [35,37]. The L-Car.HCl thermogram appears to indicate a solid-state polymorphic change i.e. polymorphic interconversion from one crystalline form to another. The enthalpy (ΔH) values were also characteristic of first-order thermodynamic phase transition (i.e. melting). It was evident that the enthalpies of melting were much higher for L-Car than for L-Car.HCl.

Representative HSM micrographs at various temperatures (Fig. 8) enabled the direct visualization of the particles as a function of temperature and confirmed the phase transitions of the formulated particles. It also demonstrated the retention of crystallinity for particles at room and physiological temperatures. The HSM confirmed the crystallinity of the drugs by showing characteristic birefringence under the cross polarized lens, in excellent agreement with the DSC and XRPD results. Also, the images were in good agreement with the melting temperatures shown in the DSC thermograms. However, that polymorphic change was not clearly seen by HSM. The ATR-FTIR (Figs. 9 and 10) spectra showed that the most intense peak on the spectra was in good agreement with the literature [37]. The characteristic peaks of L-Car, L-Car.HCl and their SD systems were identified at 1580 cm−1, 1383 cm−1, 1190 cm−1, and 625 cm−1 which corresponded to vibrations of COO− groups. Vibrations corresponding to CN group were identified at 967 cm−1, 946 cm−1, and 774 cm−1. The quantification of residual water content by KFT showed results acceptable for DPIs. However, it was clearly seen that both L-Car and L-Car.HCL were very hygroscopic and adsorbed moisture from the environment within seconds of exposure. Although both are hygroscopic, L-Car.HCl appeared to be less than the non-salt L-Car.

The *in vitro* aerosol dispersion performance (Fig. 11) was completed in this study using two FDA-approved human devices. In Table 7, SD L-Car had more optimal aerosol dispersion parameters using the Handi-Haler® at 50% PR, although the ED was better using the NeoHaler™. The ED of the SD L-Car.HCl formulations was similar. However, optimal aerosolization was achieved using the NeoHaler™ DPI device with the SD L-Car.HCl 75% PR, as shown in Table 7. Hygroscopicity of L-Car was observed and less so with L-Car HCl salt. In all these formulations, the structural cohesion and aggregation due to interparticulate interactions (i.e. van der Waals forces, capillary forces, electrostatic forces, and mechanical interlocking) clearly influenced the aerosol dispersion performance and parameters of the powder leading to relatively low ED, FPF, and RF values. It would be of future interest to examine antihygroscopic excipients molecularly mixed with L-Car and L-Car HCl salt, in addition to encapsulation approaches to reduce hygroscopicity.

**Fig. 20.**
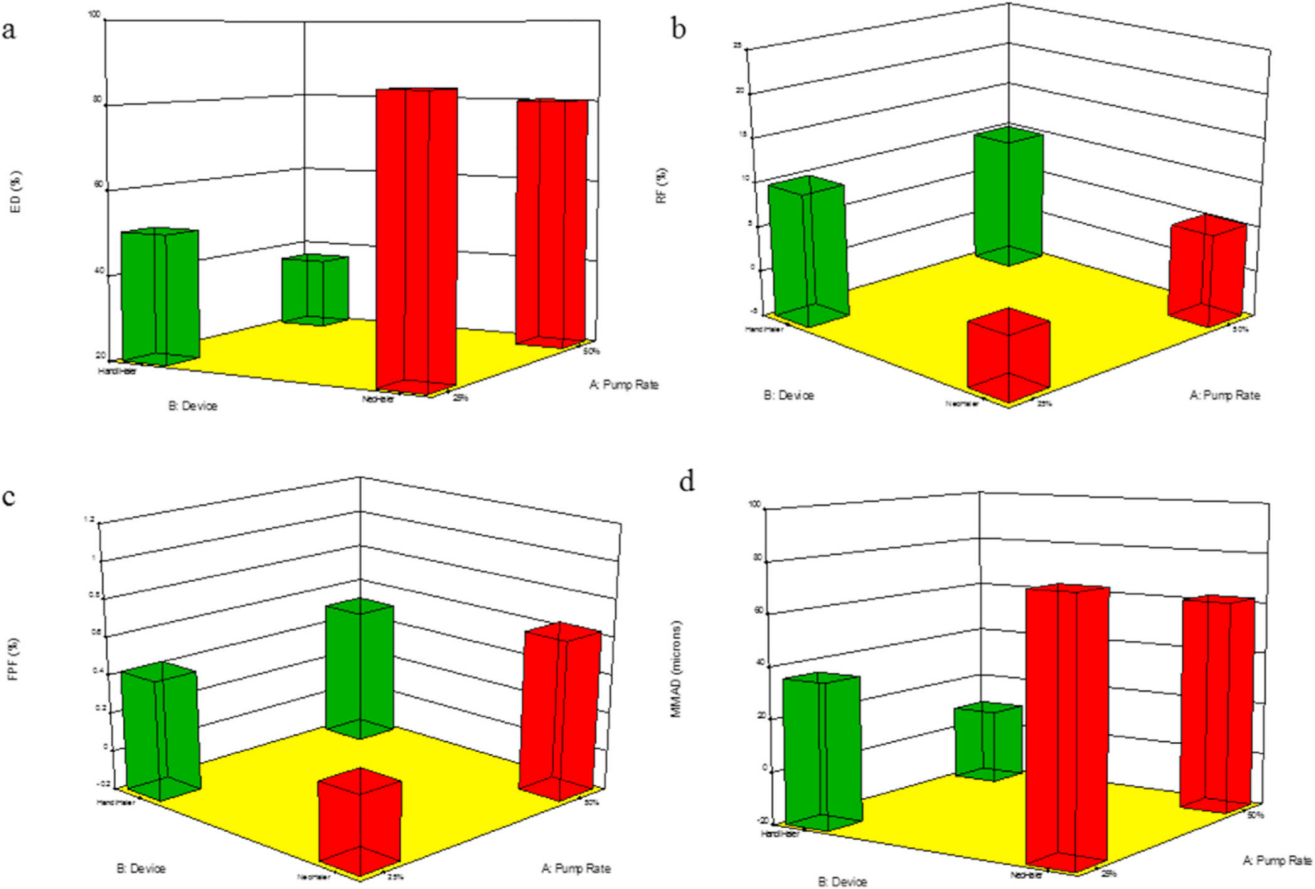
3D surface response plots showing the interplay of spray drying pump rate and the DPI device influences on the *in vitro* aerosol dispersion parameters for SD L-Car aerosolized powders for: a). ED; b). RF; c). FPF; and d). MMAD.

**Fig. 21.**
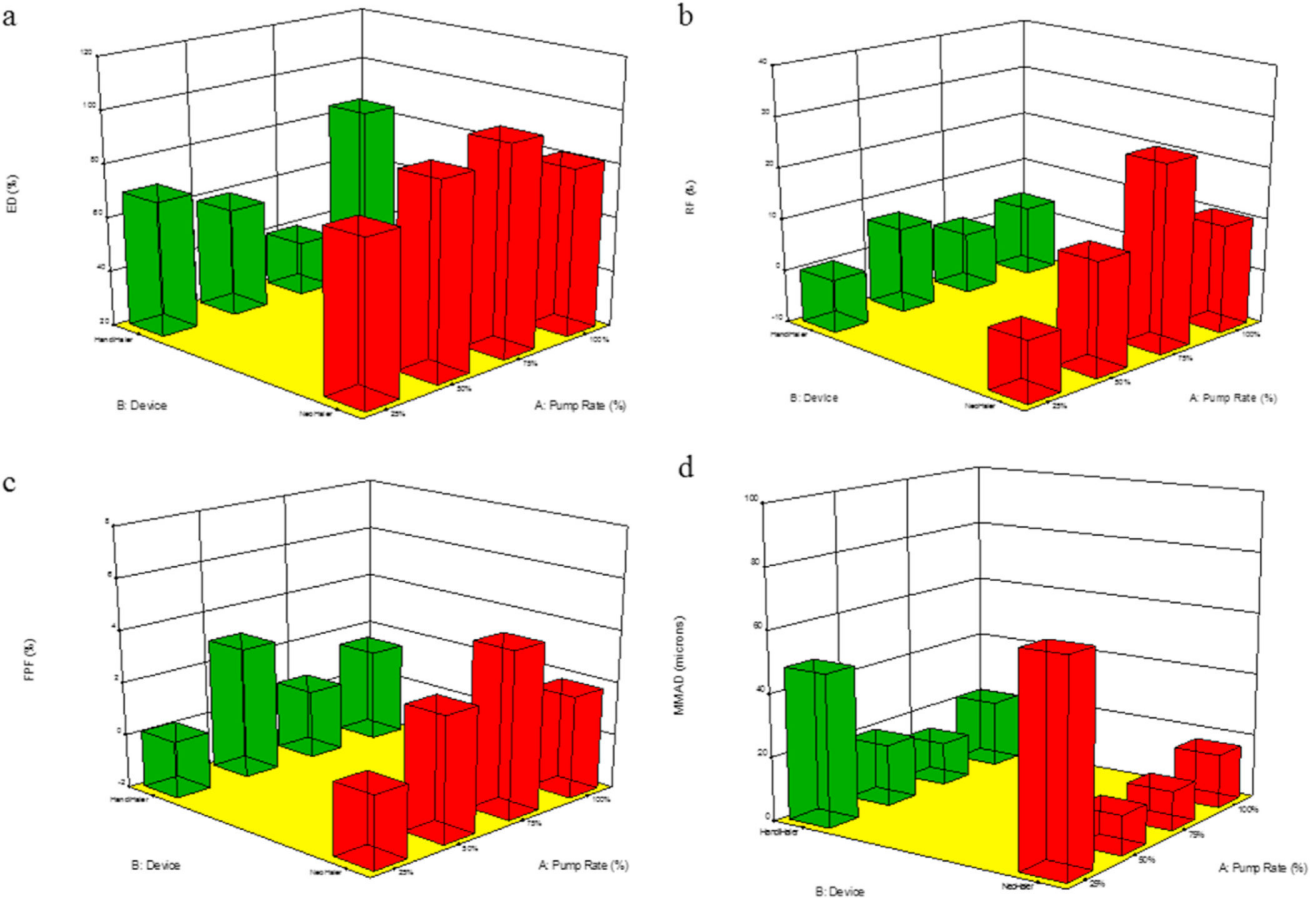
3D surface response plots showing the interplay of spray drying pump rate and the DPI device influence on the *in vitro* aerosol dispersion parameters for SD L-Car HCl aerosolized powders for: a). ED; b). RF; c). FPF; and d). MMAD.

Statistical analysis carried out using Design-Expert® showed the interplay of the spray drying pump rate effect and the inhaler device effect and influenced the aerosol dispersion parameters. For *in vitro* aerosol performance of SD L-Car systems, the 3-D plots (Figs. 20 and 21) showed that the ED was better using the NeoHaler™ device and there was a statistically significant difference between both devices (p < 0.0008), favoring the NeoHaler™. The RF for L-Car systems was not statistically significantly different (p = 0.4487). It was the same case regarding FPF, there was no statistically significant difference (p = 0.4560). The last parameter analyzed was the MMAD. In this case there was a statistically significant difference between the two parameters analyzed (p = 0.0096). For the *in vitro* aerosol dispersion performance analysis of the SD L-Car.HCl systems, there was a statistically significant difference in the ED between the two devices (p = 0.0083), favoring the NeoHaler™ device at 75% PR. The RF and the FPF parameters also showed statistically significant differences (p = 0.002 and p = 0.0314, respectively), also favoring the NeoHaler™ at 75% PR. The MMAD values showed a statistically significant difference (P < 0.0001). This difference was given by the MMAD value of SD L-Car (25% PR) using the NeoHaler™ which was showing the highest value.

The *in vitro* cell assays showed that L-Car in both forms was safe with a decrease in viability on the different cell lines being observed only at very high concentrations. The cell monolayer was also disrupted right after the administration of the high concentration solution of L-Car but covered to control levels over time.

Unfortunately, the *in vivo* test could not be carried out with the formulated dry powders due to suboptimal aerosol performance. However, liquid aerosols of L-Car clearly demonstrated that this drug was effective in reducing the development of PH. The promising findings from the *in vivo* studies serve as a proof-of-concept justifying further efforts in optimizing the dry powders. MCT rats exhibited a significant elevation in the RVP 4-weeks after MCT exposure indicating the development of severe PH. However, two weeks of once-daily inhalation aerosol treatment with L-Car, the RVSP of the rats was maintained at the near-normal value of 25 mm Hg. The weight *vs.* time plots also confirmed the state of the disease of the rats. Weight loss was significant in the MCT-induced PH rats (Fig. 17) who did not received inhaled L-Car aerosols. Moreover, some rats died in the middle of the study because of the severity of the disease. When dissecting the rats and comparing the right heart of the MCT rats *vs.* the healthy and the L-Car inhalation aerosol-treated PH rats, there was a large difference in the size of the hearts; this was reflected in a reduction in the Fulton Index. Interestingly, it was also demonstrated that there was no statistically significant difference between the L-Car plasma concentrations between the control and inhaled L-Car aerosol-treated rats indicating that the inhaled L-Car aerosols were remaining in the lung (i.e. high lung retention of L-Car) with no apparent translocation into the systemic circulation. Thus, aerosolized delivery of L-Car could be a safe and effective therapy for attenuating the progression of PH. Finally, it is important to note that L-Car as an endogenous factor in the blood and was present in all samples.

## 5. Conclusions

The study was completed as designed. Dry powder inhalers of L-Car and L-Car.HCl were successfully developed. The comparison between the physicochemical properties of the SD powders of the different forms of L-Car was successfully achieved. The extensive physicochemical characterization helped with conclusions with regard to the *in vitro* aerosol dispersion performance. In this systematic and comprehensive study, a targeted treatment for PH was successfully demonstrated and new findings on the properties of L-Car vs L-Car HCl salt were discovered.

The SD L-Car and SD L-Car HCl powders all aerosolized with all devices tested. However, there were significant interparticulate interactions leading to the nanoaggregation of the powders which reduced the aerosol dispersion parameters. L-Car was more hygroscopic than L-Car HCl; however both exhibited a certain level of hygroscopicity. Even though the aerosol performance as DPIs was unexpectedly not optimal, it was demonstrated for the first time the important proof-of-concept that inhaled L-Car aerosols are efficacious in the effective treatment of PH efficiently and in a targeted manner directly and non-invasively to the lungs. Molecularly mixing antihygroscopic excipients with L-Car and L-Car HCl salt, in addition to encapsulation approaches to reduce hygroscopicity, would be a strategy for improving the performance of the dry powder and allowing further development of this promising approach for PH treatment. A severe model of PH in rats was investigated and after only two weeks of treatment disease was reversed to near normal levels. Furthermore, it was demonstrated that L-Car and L-Car HCl were not cytotoxic on human 2D human pulmonary cell lines and on 3D human pulmonary primary cells over a wide dose range. Inhaled L-car aerosols were found to be safe *in vivo* and is a promising strategy to effectively treat and reverse this fatal disease non-invasively in a targeted manner.

## Figures and Tables

**Table 1:** Spray drying parameters using advanced spray drying in closed-mode.

Parameter	Value or Type

Inlet Temperature Aspirator rate	150 °C 100% (40 m^3^/h)
Pump rate	25% (7.5 mL/min) 50% (15 mL/min) 75% (22.5 mL/min) 100% (30 mL/min)
Gas Flow	670 L/h (55 mm Hg)
Feed Solution Concentration	0.5% w/v
Solvent	Methanol
Atomizer and Drying gas	Nitrogen
Nozzle type and diameter	Stainless steel (0.7 mm)

Spray drying parameters using advanced spray drying in closed-mode.

**Table 2:** Spray drying outlet temperatures for each spray drying pump rate (PR) condition.

System Composition	Outlet T (°C)

L-Carnitine	
SD L-Car (25% PR)	75–78
SD L-Car (50% PR)	62–65
L-Carnitine HCl	
SD L-Car HCl (25% PR)	65–68
SD L-Car HCl (50% PR)	65
SD L-Car HCl (75% PR)	45
SD L-Car HCl (100% PR)	40

**Table 3:** Particle sizing using image analysis of SEM micrographs (n ≥ 100 particles).

System	Mean (μm)	Range (μm)

**L-Carnitine**		
SD L-Car (25% PR)	15.2 ± 5.2	7.4–33.1
SD L-Car (50% PR)	12.6 ± 4.8	5.5–28.1
**L-Carnitine HCl**		
SD L-Car HCl (25% PR)	5.5 ± 1.8	2.4–10.0
SD L-Car HCl (50% PR)	5.1 ± 1.6	2.0–9.7
SD L-Car HCl (75% PR)	4.7 ± 1.5	1.4–10.0
SD L-Car HCl (100% PR)	5.4 ± 1.6	2.5–9.1

**Table 4:** DSC thermal analysis of L-Car (n = 3, mean ± SD)

L-Carnitine		

Endotherm System	Tpeak (°C)	Enthalpy (J/g)
Raw L-Car	191.26 ± 1.37	524.83 ± 139
SD L-Car (25% PR)	195.1 ± 1.61	580.73 ± 66
SD L-Car (50% PR)	193.61 ± 1.19	598.83 ± 12.67

**Table 5:** DSC thermal analysis of L-Car HCl (n = 3, mean ± SD)

L-Carnitine HCl				

System	Endotherm 1		Endotherm 2	
		
	Tpeak (°C)	Enthalpy (J/g)	Tpeak (°C)	Enthalpy (J/g)

Raw L-Car HCl	68.8 ± 0.17	5.78 ± 0.09	139 ± 1.24	79.4 ± 10.44
SD L-Car HCl (25% PR)	68.33 ± 0.09	4.99 ± 0.46	130.72 ± 0.52	53.48 ± 8.82
SD L-Car HCl (50% PR)	68.35 ± 0.12	5.29 ± 0.41	132.55 ± 1.09	63.98 ± 6.40
SD L-Car HCl (75% PR)	68.54 ± 0.04	5.74 ± 0.75	134.43 ± 0.71	73.46 ± 3.97
SD L-Car HCl (100% PR)	68.56 ± 0.11	5.17 ± 0.21	135.40 ± 0.37	76.94 ± 0.95

DSC thermal analysis of L-Car HCl (n = 3, mean ± SD).

**Table 6:** Residual water content for SD dry powder inhalation formulations as quantified analytically by coulometric KFT (n = 3, mean ± SD).

System	Residual Water Content % (w/w)

**L-Carnitine**	
Raw L-Car	2.68 ± 0.80
SD L-Car (25% PR)	2.78 ± 1.19
SD L-Car (50% PR)	3.02 ± 1.06
**L-Carnitine HCl**	
Raw L-Car HCl	0.45 ± 0.09
SD L-Car HCl (25% PR)	2.69 ± 0.51
SD L-Car HCl (50% PR)	1.06 ± 0.35
SD L-Car HCl (75% PR)	0.80 ± 0.11
SD L-Car HCl (100% PR)	1.07 ± 0.37

**Table 7:** In Vitro aerosol dispersion performance parameters using the next generation Impactor™ for SD aerosol systems including fine particle fraction (FPF), respirable fraction (RF), and emitted dose (ED). (n = 3, mean ± SD).

System Composition	ED (%)	FPF (%)	RF (%)	MMAD (μm)	GSD

**L-Carnitine NeoHaler**™					
SD L-Car (25% PR)	85.10 ± 14.43	0.21 ± 0.06	2.22 ± 1.26	76.92 ± 9.28	3.09 ± 0.78
SD L-Car (50% PR)	80.46 ± 4.92	0.65 ± 0.5	5.66 ± 6.22	62.8 ± 29.38	2.91 ± 1.27
**L-Carnitine HandiHaler**®					
SD L-Car (25% PR)	50.57 ± 7.60	0.44 ± 0.29	10.20 ± 7.17	36.31 ± 20.91	2.78 ± 0.76
SD L-Car (50% PR)	37.11 ± 9.38	0.53 ± 0.32	10.32 ± 9.77	8.4 ± 8.56	1.76 ± 0.94
**L-Carnitine HCl NeoHaler**™					
SD L-Car HCl (25% PR)	80.23 ± 25.97	0.76 ± 0.18	1.87 ± 0.86	66.52 ± 21.74	3.5 ± 0.08
SD L-Car HCl (50% PR)	92.45 ± 5.53	2.78 ± 0.52	12.05 ± 6.55	12.86 ± 4.23	1.49 ± 0.32
SD L-Car HCl (75% PR)	98.22 ± 0.90	4.4 ± 0.45	26.39 ± 13.06	12.5 ± 2.47	1.79 ± 0.15
SD L-Car HCl (100% PR)	82.84 ± 17.7	1.94 ± 0.52	11.08 ± 0.52	17.38 ± 3.98	1.81 ± 0.22
**L-Carnitine HCl HandiHaler**®					
SD L-Car HCl (25% PR)	70.61 ± 16.62	0.21 ± 0.14	0.45 ± 0.29	49.14 ± 13.58	3.36 ± 0.41
SD L-Car HCl (50% PR)	60.51 ± 6.33	3.08 ± 2.3	6.84 ± 6.65	19.95 ± 5.58	2.38 ± 1.05
SD L-Car HCl (75% PR)	40.3 ± 2.17	0.68 ± 0.55	2.09 ± 1.88	13.98 ± 3.22	1.55 ± 0.30
SD L-Car HCl (100% PR)	85.42 ± 3.04	1.6 ± 0.75	4.02 ± 2.46	21.82 ± 8.8	1.69 ± 0.56

**Table 8:** RSVP and Fulton Index measurements for the control groups and in the MCT-rat model of PH in male Sprague Dawley rats (n = 5, Mean ± SD).

	Control Group: Vehicle	Control Group: Inhaled Carnitine	MCT-Inhaled Vehicle Group	MCT-Inhaled Carnitine Group

**RVSP (mm Hg)**	24.50 ± 0.89	25.44 ± 0.57	43.71 ± 2.94 (p < 0.05)	34.29 ± 2.34 (p < 0.05)
**Fulton Index**	0.26 ± 0.01	0.26 ± 0.01	0.35 ± 0.01	0.31 ± 0.01
